# Supplementary material for: Observational study of the implementation of telephone advice nursing in Sweden: did callers follow recommendations and did the rate of healthcare visits change?
Source: BMJ Open. 2021 Aug 19;11(8):e051233. doi: 10.1136/bmjopen-2021-051233 (PMC8378364; doi:10.1136/bmjopen-2021-051233)
Supplement: Author's manuscript [file bmjopen-2021-051233.draft_revisions.pdf]

BMJ Open is committed to open peer review. As part of this commitment we make the peer review history of every article we publish publicly available.

When an article is published we post the peer reviewers' comments and the authors' responses online. We also post the versions of the paper that were used during peer review. These are the versions that the peer review comments apply to.

The versions of the paper that follow are the versions that were submitted during the peer review process. They are not the versions of record or the final published versions. They should not be cited or distributed as the published version of this manuscript.

BMJ Open is an open access journal and the full, final, typeset and author-corrected version of record of the manuscript is available on our site with no access controls, subscription charges or pay-per-view fees (http://bmjopen.bmj.com).

If you have any questions on BMJ Open's open peer review process please email info.bmjopen@bmj.com

BMJ Open

The implementation of telenursing in Sweden: an observational study focusing on whether callers follow recommendations, and if there is there a change in the rate of healthcare visits

Journal:	*BMJ Open*
Manuscript ID	bmjopen-2021-051233
Article Type:	Original research
Date Submitted by the Author:	13-Mar-2021
Complete List of Authors:	Sundberg, Amanda; Karolinska Institute Department of Learning Informatics Management and Ethics<br>Wahlberg, Anna Carin; Karolinska Institutet Institutionen för neurobiologi vårdvetenskap och samhälle, Department of Neurobiologi<br>Zethraeus, N; Karolinska Institute Department of Learning Informatics Management and Ethics<br>Karampampa, Korinna; Karolinska Institute Division of Insurance Medicine, Department of Clinical Neuroscience
Keywords:	Telemedicine < BIOTECHNOLOGY & BIOINFORMATICS, HEALTH SERVICES ADMINISTRATION & MANAGEMENT, PRIMARY CARE, HEALTH ECONOMICS, Organisation of health services < HEALTH SERVICES ADMINISTRATION & MANAGEMENT

SCHOLARONE™
Manuscripts

I, the Submitting Author has the right to grant and does grant on behalf of all authors of the Work (as defined in the below author licence), an exclusive licence and/or a non-exclusive licence for contributions from authors who are: i) UK Crown employees; ii) where BMJ has agreed a CC-BY licence shall apply, and/or iii) in accordance with the terms applicable for US Federal Government officers or employees acting as part of their official duties; on a worldwide, perpetual, irrevocable, royalty-free basis to BMJ Publishing Group Ltd ("BMJ") its licensees and where the relevant Journal is co-owned by BMJ to the co-owners of the Journal, to publish the Work in this journal and any other BMJ products and to exploit all rights, as set out in our licence.

The Submitting Author accepts and understands that any supply made under these terms is made by BMJ to the Submitting Author unless you are acting as an employee on behalf of your employer or a postgraduate student of an affiliated institution which is paying any applicable article publishing charge ("APC") for Open Access articles. Where the Submitting Author wishes to make the Work available on an Open Access basis (and intends to pay the relevant APC), the terms of reuse of such Open Access shall be governed by a Creative Commons licence – details of these licences and which Creative Commons licence will apply to this Work are set out in our licence referred to above.

Other than as permitted in any relevant BMJ Author's Self Archiving Policies, I confirm this Work has not been accepted for publication elsewhere, is not being considered for publication elsewhere and does not duplicate material already published. I confirm all authors consent to publication of this Work and authorise the granting of this licence.

For peer review only

Title:

The implementation of telenursing in Sweden: an observational study focusing on whether callers follow recommendations, and if there is there a change in the rate of healthcare visits

Authors:

Amanda Sundberg[1], Anna Carin Wahlberg[2], Niklas Zethraeus[3], Korinna Karampampa[4]

Affiliations:

[1] Employee at Inera AB, Stockholm, Sweden, and Alumni of Master of Medical Science with a Major in Public Health Scienes from the Department of Learning, Informatics, Management and Ethics, Karolinska Institutet, Stockholm, Sweden

[2] Division of Nursing, Department of Neurobiology, Care Sciences and Society, Karolinska Institutet, Stockhom, Sweden

[3] Department of Learning, Informatics, Management and Ethics, Karolinska Institutet

[4] Division of Insurance Medicine, Department of Clinical Neuroscience, Karolinska Institutet, Stockholm, Sweden

Corresponding author:

For peer review only

Korinna Karampampa

Division of Insurance Medicine,

Department of Clinical Neuroscience,

Karolinska Institutet, Stockholm, Sweden

Email: Korinna.karampampa@ki.se

For peer review only

Abstract

Objectives: Telenursing is introduced in many countries with one key aim being a reduction of avoidable healthcare visits. The aim of this study was to explore whether callers to a telenursing service followed the telenurses' recommendations, and whether there was a change in the level and trend of the rate of healthcare visits after the introduction of telenursing.

Design: Observational study.

Setting: Primary and secondary care in Jönköping Region, Sweden.

Participants: Primary outcome: telenursing calls, 06-23 hours, 2014-2015 (n=185,994) and any subsequent outpatient healthcare visits within 72 hours of each call. Secondary outcome: outpatient healthcare visits 2012-2015 (n=6,877,266).

Primary outcome: Proportion of callers who visited healthcare within the time period advised by the telenurse.

Secondary outcome: Change in level or trend of the overall rate of healthcare visits per 1,000 persons and four-week period after the introduction of telenursing, with subgroup analysis for primary and secondary care.

Results: 77 % of callers who were recommended either to visit healthcare within 24 hours or to 'wait and see' followed the recommendations. There was no significant change in level (-5.15; 95% CI -15,80 to 5,50; *p*=0.349) or trend (-0.24; 95% CI -0,86 to 0,38; *p*=0.448) of the overall rate of visits per 1,000 persons and four-week period after the introduction of telenursing. For the rate of primary care visits, an

For peer review only

increase in level (8.01; 95% CI 6,36 to 9,66; *p*<0.001) and trend (1.28; 95% CI 1,17 to 1,39; *p*<0.001) were observed. For the rate of secondary care visits, a decrease in level (-8.77, 95% CI -14,41 to -3,13; *p*=0.004) and trend (-1.03, 95% CI -1,35 to -0,71; *p*<0.001) were observed.

Conclusions: The introduction of telenursing may have contributed to a shift in the rate of healthcare visits from secondary to primary care.

Keywords: Telephone advice nursing, Telenursing, Telehealth, Interrupted time series analysis

Strengths and limitations of this study

- *Study based on complete registry data with over 7 million observations.*
- *Statistical analysis using interrupted time series analysis with segmented regression.*
- *No non-equivalent comparison was used to control for potential confounders in the interrupted time series analysis.*
- *Results might not be generalizable to the rest of Sweden, or to other countries*

For peer review only

Background

A striking development around the Western world is the increased availability of telephone triage and advice services (henceforth telenursing), where telenurses provide callers with triage, advice, support, education, referral to adequate care levels, healthcare information and coordinate healthcare resources.[1] Telenursing has been implemented in the United Kingdom, Denmark, Sweden, Australia, the USA, the Netherlands and Canada.[2–4] In Sweden, the telenursing service 1177 Vårdguiden (henceforth 1177) was implemented successively across the regions between 2003-2013.[5] The service has the five minimum components required to ensure patient safety: qualified staff, medically approved guidelines, electronic medical records, staff training programmes and standards.[6,7]

One key aim from policymakers when implementing telenursing services is helping care seekers find the appropriate level of care and reducing avoidable healthcare visits.[4,8,9] Swedish healthcare providers hypothesised that 1177 would contribute to a more efficient healthcare system.[10–12] It was predicted that if callers followed the telenurses' recommendations, the service would impact upon the rates of healthcare visits as a greater proportion of patients would be able to self-care or seek care at more appropriate healthcare levels than they would without access to telenursing.[10] The anticipated effect from this transition was an overall reduction of costly emergency visits to secondary care, which would free resources in the healthcare system. The rate of primary care visits was expected to remain more or less unchanged but with a more appropriate case mix.[10]

For peer review only

The available evidence worldwide regarding the extent to which callers follow recommendations from telenurses and telenursing's impact on healthcare visits is inconsistent, suggesting that context-specific factors such as healthcare organization affect the external validity of findings.[2,4,8] Identifying key policy lessons for any given telenursing service therefore requires it to be studied specifically.

For 1177, evidence regarding the extent to which callers follow recommendations from telenurses is diverse. 1177's own surveys to callers find that 90 % follow the recommendations,[13] while a government agency used observational data to find that 55 – 78 % follow recommendations depending on the advice provided.[14]

Similarly, evidence regarding 1177's impact on the rate of healthcare visits is scarce. Four studies have found telenursing services in Sweden to redistribute visits to less costly levels of care.[15–18] Two of the studies concerned local predecessors to 1177.[15,16] All studies based their conclusions on self-reported survey data rather than observational data, which possibly limits the strength of the evidence.

To explore the hypothesis that 1177 may have freed resources in the healthcare system through reducing avoidable healthcare visits, the aim of this study was to use observational data to evaluate whether 1177 callers follow the telenurses' recommendations, and whether there was any change in the level and trend of healthcare visits after the service was introduced.

For peer review only

Methods

Study cohort and setting

This study was based on patient-level data from the patient administrative system in the Region of Jönköping, Sweden. The region manages a tax-financed healthcare system with universal coverage for its 345,000 inhabitants.[19–22] 1177 was implemented September 2nd 2013. Before that, telenursing was provided by individual hospitals and health care centres in the region. When 1177 was introduced, seven regional telephone advice lines were closed, including those to emergency departments and out-of-hours centres. 1177 was intended as a first point of contact with healthcare for inhabitants, along with the options of reading information on the 1177 website, calling a healthcare centre or visiting an emergency department or out-of-hours care centre.[23]

To determine the percentage of callers who follow the recommendations of the telenurse, calls to 1177 were included in the study and linked to information on any subsequent outpatient healthcare visits (emergency visits, visits that required referrals and visits that did not require referrals). The linkage and subsequent anonymization was performed by the system administrator using the unique personal identity number that Swedish residents have. Only anonymized patient-level data was used in the study.

In addition, anonymized patient-level data on all outpatient healthcare visits (emergency visits, visits that required referrals and visits that did not require referrals) in the region was used to examine whether there was any change in the level and trend of healthcare visits after 1177 was introduced.

For peer review only

The study was approved by the Swedish Ethical Review Authority, registered under number 2019-01784.

Definitions and description of analysis for the study outcomes

The first study outcome of whether callers followed recommendations of the telenurse was defined as the proportion of callers who visited healthcare within the time period advised by the telenurse.

To evaluate this outcome, calls to 1177 between 6 am and 11 pm and any subsequent healthcare visit at any time of day, that took place within 72 hours of the call between January 1st 2014 and December 31st 2015 (n = 185,994) were used. Visits by patients that were not registered as residents of the region and calls that referred patients to healthcare outside of the region were excluded.

Variables included date and time of call, callers' age and sex, the 'urgency level', as well as the name of any healthcare unit visited within 72 hours of the call, and if the visit occurred within 24 hours.

The variable 'urgency level' described how urgent the telenurse thought it was for the caller to visit healthcare. It had five levels: 'immediately', 'quickly' (meaning 'within a few hours'), 'within 24 hours', 'a weekday in near time' (meaning within a week or a few weeks) and 'wait and see' (meaning no need to visit healthcare). These five levels were collapsed into the three levels, since the data did not allow following the caller's subsequent behaviour on a more granular level:

For peer review only - http://bmjopen.bmj.com/site/about/guidelines.xhtml

- *Visit within 24 hours (including 'immediately', 'quickly' and 'within 24 hours')*
- *Visit within a few weeks (including 'a weekday in near time')*
- *'Wait and see' (including 'wait and see')*

The urgency level assigned to each call was mapped to the data on healthcare visits, providing the proportion of callers that were recommended to visit within 24 hours and did so, as well as the proportion of callers recommended to 'wait and see' that did not visit healthcare within 72 hours of the call. The proportion of callers who followed the recommendation to visit healthcare within a few weeks could not be determined as the data on healthcare visits only stretched to 72 hours after the call.

To evaluate the second study outcome, i.e. to determine whether the rate of healthcare visits changed after the introduction of 1177, all outpatient healthcare visits between January 23, 2012 and December 20, 2015 were used (n=6,877,266). The data covered 51 four-week periods; 21 before 1177 was implemented and 30 after. Variables included date and time of the visit and the name of the healthcare unit visited.

Interrupted time series with segmented regression was used to study this outcome. The series' data points were calculated as visits per 1,000 population and four-week period. The rates were controlled for population growth.

Three time series were made, one for all outpatient visits (primary and secondary care) and one for each subgroup of primary and outpatient secondary care visits. To create the subgroups for outpatient primary and secondary care, the healthcare units were

For peer review only

categorised according to a definition from the Swedish Agency for Health and Care Services:[14]

- *Primary care: primary care centres, physicians' centres, out-of-hours primary care, children's healthcare centres and maternal healthcare centres.*
- *Secondary care: specific or hospital-related specialist surgeries including emergency care.*

Regression models with two segments were fitted to the data, one for the period pre-1177 and one for the period after. Separate levels (intercepts) and trends (slopes) were estimated for each segment.[24] Statistical tests were carried out to estimate changes in the level or trend after the introduction of 1177. To estimate the size of the effect, the models were used to calculate the rates one year (12 four-week periods) after telenursing was introduced and the relative changes compared to the rates that would have been if the trend before telenursing was introduced had continued unchanged.

To control for seasonality, harmonic sine and cosine pairs ('Fourier terms') were added to the model.[25] To control for autocorrelation, a moving averages term of order q=13 was added using generalized least squares regression with a maximum likelihood method. Three plausible alternative autocorrelation terms were tested using likelihood-ratio tests. Since the akaike information criterion did not indicate improvements to the fit of the model, q=13 was retained. Nevertheless, some autocorrelation remained.

For peer review only

For peer review only - http://bmjopen.bmj.com/site/about/guidelines.xhtml

Results

Calls to the telenursing service represented 5 % of outpatient contacts. The proportion that followed the recommendation differed between groups (Table 1). A majority of those recommended to visit healthcare within 24 hours did so (73 %), and 84 % of those recommended to 'wait and see' did not visit healthcare within 72 hours. Combining these groups, 77 % followed the recommendation. When stratified by age groups and sex, the proportion that followed the telenurses' recommendation varied between 66 % (females aged 20-39) and 90 % (children aged 0-9).

Table 1. Callers that followed the recommendation to visit healthcare or 'wait and see', Jönköping Region 2014-2015.

		Followed the recommendation to visit healthcare within 24 h n(%)		Followed the recommendation to 'wait and see' n(%)		Both groups combined n(%)	
	All (n=162,614)	75,159	(73)	50,836	(84)	125,995	(77)
	Males (n=72,180)	34,403	(75)	22,533	(86)	56,936	(79)
	Females (n=90,434)	40,756	(72)	28,303	(83)	69,059	(76)
All	Age group	Followed the recommendation to visit healthcare within 24 h n(%)		Followed the recommend-ation to 'wait and see' n(%)		Both groups combined n(%)	
	0-9 (n=51,412)	18,925	(70)	22,045	(90)	40,970	(80)
	10-19 (n=16,032)	8,559	(79)	4,388	(84)	12,947	(81)
	20-39 (n=43,489)	18,605	(68)	13,273	(82)	31,878	(73)
	40-59 (n=25,289)	13,972	(77)	5,624	(80)	19,596	(77)
	>60 (n=26,392)	15,098	(79)	5,505	(77)	20,603	(78)
Males	Age group						
	0-9 (n=27,562)	10,246	(71)	11,769	(90)	22,015	(80)
	10-19 (n=7,365)	4,065	(81)	2,857	(84)	6,044	(82)
	20-39 (n=15,488)	7,232	(71)	4,496	(84)	11,728	(76)
	40-59 (n=10,706)	6,223	(78)	2,207	(80)	8,430	(79)
	>60 (n=11,059)	6,637	(80)	2,082	(76)	8,719	(79)
Females	Age group						
	0-9 (n=23,850)	8,679	(70)	10,276	(90)	18,955	(79)
	10-19 (n=8,667)	4,494	(78)	2,409	(83)	6,903	(80)
	20-39 (n=28,001)	11,373	(66)	8,777	(81)	20,150	(72)
	40-59 (n=14,583)	7,749	(76)	3,417	(79)	11,166	(77)
	>60 (n=15,333)	8,461	(78)	3,423	(77)	11,884	(78)

For peer review only

For peer review only - http://bmjopen.bmj.com/site/about/guidelines.xhtml

Overall, for all healthcare visits, the final segmented regression model showed no significant changes in level (-5.15; 95% CI -15.8 to 5.5; *p*=0.349) or trend (-0.24; 95% CI -0.86 to 0.38; *p*=0.448) of the healthcare visits after the introduction of 1177. Statistically significant changes after the introduction of 1177 were found only in the subgroup analysis. Immediately after the introduction of 1177, primary care visits had an increase of 8.01 visits per 1,000 persons (95% CI 6.36 to 9.66; *p*<0.001) and had a trend increase of 1.28 visits per 1,000 per four-week period (95% CI 1.17 to 1.39; *p*<0.001) In contrast, visits to secondary care decreased with 8.77 visits per 1,000 weeks immediately after the introduction of telenursing (95% CI -14.41 to -3.13; *p*=0.004), and had a trend decrease of 1.03 visits per 1,000 per four week period (% CI -1.35 to -0.71; *p*<0.001) (Table 2 and Figure 1).

When the regression models were used to estimate the effect size one year (12 four-week periods) after the introduction of 1177, an 11.5 % increase in the rate of primary care visits and an 11 % decrease in the rate of secondary care visits was found (Table 3).

Table 2. Results of the interrupted time series analysis for healthcare visits, Jönköping Region 2012-2015.

	Level (intercept)	Trend (slope)	Change in level after telenursing	Change in trend after telenursing
All visits (primary and secondary care)				
Co-efficient	376.909	0.769	-5.152	-0.244
95% CI	368.501 to 385.317	0,058 to 1.480	-15.797 to 5.497	-0.863 to 0.383
p-value	<0.001	0.040	0.349	0.448
Primary care visits				
Co-efficient	240.49	-1.164	8.013	1.279
95% CI	238.738 to 242.242	-1.297 to -1.031	6.36 to 9.66	1.17 to 1.39
p-value	<0.001	<0.001	<0.001	<0.001
Secondary care visits				
Co-efficient	142.861	1.437	-8.767	-1.031

For peer review only

For peer review only - http://bmjopen.bmj.com/site/about/guidelines.xhtml

95% CI	138.594 to 147.128	1.074 to 1.8	-14.413 to -3.127	-1.349 to -0.711
p-value	<0.001	<0.001	0.004	<0.001

Figure 1. Interrupted time series analysis for the rate of healthcare visits, Jönköping Region 2012-2015. (a) All visits (primary and secondary care). (b) Primary care visits. (c) Secondary care visits.

-------------*Insert Figure 1*-------------

Table 3. Observed and counterfactual rates of healthcare visits 12 four-week periods after telenursing was introduced.

	All visits (primary and secondary care) (p>0.05)	Primary care visits (p<0.05)	Secondary care visits (p<0.05)
Observed rate per 1,000 persons	394.20	225.59	169.13
Counterfactual rate per 1,000 persons	402.28	202.23	190.27
Absolute change (observed – counterfactual rate)	-8.02	23.36	-21.14
Relative change	-2.01 %	11.55 %	-11.11%

For peer review only

Discussion

Together, the findings of this study provide some support to the hypothesis that introducing 1177 contributed to improving healthcare system efficiency. A prerequisite for 1177 to be able to impact the rates of healthcare visits is that callers follow the recommendations, and this study found that a large proportion did so (77 %). This study also found an increase in the rate of healthcare visits to primary care and a decrease in the rate of visits to secondary care after the introduction of 1177. It is possible that the introduction of 1177 contributed to these changes, enabling more patients to seek care at lower levels than they could prior to the service's introduction.

Compared to 1177's own survey-based findings on the proportions that follow the recommendations of the telenurse (90 %), 77 % is low.[13] This is also the case when considering earlier survey-based studies on local predecessors to 1177.[1,15,16,26] However, the finding is similar to those of a government agency report that used observational data.[14] This contrast between survey data and observational data is supported by observations of systematic reviews of the international literature on telenursing, according to which self-reported data tends provide higher proportions than observational data.[2]

These inconsistent results of survey and observational data respectively may be considered as complementary as they to some extent measure different constructs. While observational studies require researchers to define what it means to follow the recommendations of the telenurse, survey-based studies let respondents interpret its meaning subjectively. Although the latter study design suffers from threats to validity, primarily from recall-, social desirability- and nonresponse bias,[27–29] it also allows for

For peer review only

capturing nuances of what it means to follow recommendations, and for studying the patient's perspective. Consider, for example, the broad range of possible care-seeking scenarios concealed behind the proportions found in this study:

- *When callers appear to have followed the recommendations, it is not certain that the call to 1177 were what led them to do what they did. They may have based their decisions on consultations with primary care, family and friends, websites, and so forth.*
- *The data in this study only showed the telenurse's main recommendation and cannot reflect potential alternative courses of action that the telenurse and caller may have also discussed, for example depending on how the patient's health status developed.*
- *The care recommended by the telenurse may have been inaccessible to the caller, making it impossible to follow the recommendations.*
- *Callers may have chosen not to follow the recommendations because they lacked confidence in the telenurse or found the recommendations inappropriate.*[18,30,31]

The observed changes in the rate of healthcare visits broadly support the findings of previous survey-based studies in Sweden, which have suggested that telenursing services in the Swedish setting reduce emergency visits.[15–17] The interpretation that 1177 contributed to the observed changes in the rates of healthcare visits is also strengthened by the lack of plausible alternative explanations: the supply of primary and secondary care was stable throughout the study period and no other potential confounders have been identified.[19–22]

For peer review only

Strengths and limitations of the study

The strength of the study is that complete anonymized patient-level data was used, providing a strong design for answering the research questions. Unlike previous studies, this study's findings were derived from a large number of observations, free from the biases of surveys.[27] The interrupted time series analysis was the next-best method for studying the rate of healthcare visits before and after the introduction of 1177 given a randomised, controlled trial was not possible.[24,32,33]

This study also has several limitations. Ideally, a non-equivalent comparison should have been added to control for potential confounders.[24] However, as a control group was not available, media archives and grey literature were searched to identify potential confounding events, but none were found.

In the statistical analysis, the segmented regression model fitted to the data had signs of autocorrelation despite attempts to control for this. It is not known to what extent this affected the results, and these should as always be interpreted keeping the assumptions and limitations of the model in mind.

This study has focused on 1177 in one Swedish region. While the healthcare organization across the regions do not differ substantially, some differences may be present regarding the availability of healthcare services and the population demographics. Therefore, the results might not be generalizable to the rest of Sweden, or to other countries.

Implications for future research and policy

For peer review only

297 Studies have established that one of the largest challenges experienced by telenurses at 1177 in Sweden is the lack of adequate resources in the healthcare organisation, with insufficient availability of primary care being the largest problem.[6,34–39,44] Therefore, even if – as the findings in this study suggests – callers to 1177 are willing to follow the telenurses' recommendation, it is not always possible for them to do so. If the healthcare organisation is not adapted to the needs of the patients, 1177 cannot to contribute to system efficiency with its full potential. Future studies of 1177's contribution to system efficiency should treat it not as an isolated service but as one who's efficacy is interlinked with the functioning of the healthcare system as a whole.

Regarding the overall use of 1177, this study found that only 5 % of outpatient healthcare contacts were calls to 1177, raising the question of whether more patients could benefit from using the service. This aspect has not been studied in Sweden, but studies in the United Kingdom have found that the – now former – telenursing service National Health Service Direct was used more by some groups, important determinants being ethnicity, gender, age and socio-economic status.[40–43] To better understand the determinants of healthcare use, these aspects should be approached in future studies.

Conclusions

The results indicate that large proportions of those who call 1177 follow the recommendations and that the introduction of 1177 in Region Jönköping may have contributed to a shift in healthcare visits from secondary to primary care. The results provide some support to the hypothesis that 1177 may have contributed to system efficiency, freeing resources through reducing unnecessary costly healthcare visits.

For peer review only

Declarations

Ethics approval and consent to participate

Informed consent was not collected from the participants of this study. The study was approved by the Swedish Ethical Review Authority, registered under number 2019-01784.

Patient and public involvement

Patients and public were not involved in the design and conduct of this study.

Availability of data and materials

The data that support the findings of this study are available from Region Jönköping, but restrictions apply to the availability of these data, which were used under license for the current study, and so are not publicly available.

Competing interests

AS is employed by Inera AB, a limited company owned by Swedish regions, municipalities as well as the Swedish Association of Local Authorities and Regions (SALAR) that administrates the technical solutions used in 1177. AS conducted this research as part of her master's degree in Public Health Sciences, Health economics, policy and management while she was on leave of absence from her employment. The study findings have been interpreted scientifically by all co-authors, without the involvement of Inera AB, and not in the interest of 1177.

For peer review only

All other co-authors (ACW, NZ, and KK) are employed at or affiliated with Karolinska Institutet, Stockholm, Sweden.

KK is currently employed by Gilead Sciences AB, a pharmaceutical company which is not involved with 1177 in Sweden.

Funding

This research received no specific grant from any funding agency in the public, commercial or not-for-profit sectors.

Authors' Contributions

AS, NZ and KK contributed to the conceptualization of the research questions, study design and methods. AS performed the analysis of the data, interpreted the study findings, and drafted the manuscript. All remaining authors, AS, NZ, KK and ACW assisted in the interpretation of the study findings and contributed to the manuscript with comments, suggestions and text.

Acknowledgements

Not applicable.

For peer review only

References

1. Wahlberg AC. Telephone advice nursing: callers' perceptions, nurses' experience of problems and basis for assessments. Karolinska Institutet, 2004.

2. Lake R, Georgiou A, Li J, et al. The quality, safety and governance of telephone triage and advice services – an overview of evidence from systematic reviews. BMC Health Serv Res; 17. Epub ahead of print 2017. DOI: 10.1186/s12913-017-2564-x.

3. Wahlberg AC, Bjorkman A. Expert in nursing care but sometimes disrespected-Telenurses' reflections on their work environment and nursing care. J Clin Nurs 2018; 27: 4203–4211.

4. Gidora H, Borycki EM, Kushniruk AW. Effects of Telenursing Triage and Advice on Healthcare Costs and Resource Use. Stud Health Technol Inform 2019; 257: 133–139.

5. Leppänen V. Inledning. In: Telefonrådgivning inom hälso- och sjukvård. Hungary: Studentlitteratur, 2008.

6. Wheeler SQ, Greenberg ME, Mahlmeister L, et al. Safety of Clinical and Non-Clinical Decision Makers in Telephone Triage: A Narrative Review. J Telemed Telecare 2015; 21: 305–322.

7. Inera. 1177 Vårdguiden på telefon, https://www.inera.se/tjanster/1177-vardguiden-pa-telefon/ (2018, accessed 11 January 2018).

For peer review only

8. Ismail SA, Gibbons DC, Gnani S. Reducing inappropriate accident and emergency department attendances: a systematic review of primary care service interventions. Br J Gen Pr 2013; 63: e813-20.

9. Campbell JL, Fletcher E, Britten N, et al. Telephone triage for management of same-day consultation requests in general practice (the ESTEEM trial): a cluster-randomised controlled trial and cost-consequence analysis. Lancet 2014; 384: 1859–1868.

10. Landstingsförbundet. Vårdråd direkt - Sjukvårdsrådgivningar i samverkan. Slutrapport från utredningen om nationellt samordnad sjukvårdsrådgivning. Stockholm: Landstingsförbundet, 2003.

11. Stienstedt G. Effektiv vård: slutbetänkande av en nationell samordnare för effektivare resursutnyttjande inom hälso- och sjukvården. Stockholm: Statens Offentliga Utredningar, 2016.

12. Kaminsky E, Röing M, Björkman A, et al. Telephone nursing in Sweden: A narrative literature review. Nurs Health Sci 2017; 19: 278–286.

13. Eklöf P. 1177 Vårdguiden på telefon, https://www.1177.se/Om-1177/1177-sjukvardsradgivning/ (2017, accessed 12 January 2018).

14. En primär angelägenhet - kunskapsunderlag för en stärkt primärvård med patienten i centrum. 2017:3, Myndigheten för vård- och omsorgsanalys, 2017.

15. Nordling S. Ekonomisk utvärdering av sjukvårdsupplysning per telefon i Jämtland och Malmö. Lund: IHE, 2001.

For peer review only

16. Marklund B, Ström M, Månsson J, et al. Computer-supported telephone nurse triage: an evaluation of medical quality and costs. J Nurs Manag 2007; 15: 180–7.

17. Rahmqvist M, Husberg M. Effekter av sjukvårdsrådgivning per telefon - en analys av rådgivningsverksamheten 1177 i Östergötland och Jämtland. Linköping: Linköpings universitet, Centrum för utvärdering av medicinsk teknologi, 2009.

18. Martinsson J, Gustafsson S. Modeling the effects of telephone nursing on healthcare utilization. Int J Med Inf 2018; 113: 98–105.

19. Region Jönköpings län. Årsredovisning 2015: med förvaltningsberättelse. 2016.

20. Region Jönköpings län. Årsredovisning 2014: med förvaltningsberättelse. 2015.

21. Landstinget i Jönköpings län. Årsredovisning 2012: med förvaltningsberättelse. Huskvarna, 2013.

22. Landstinget i Jönköpings län. Årsredovisning 2013: med förvaltningsberättelse. Ängelholm, 2014.

23. Landstingsstyrelsen, Landstinget i Jönköpings län. Införande av 1177 och ett gemensamt kundcenter. Missiv LJ 2012/82, 22 August 2012.

24. Shadish WR, Cook TD, Campbell DT. Experimental and Quasi-Experimental Designs for Generalized Causal Inference. Belmont, California: Wadsworth Cengage Learning, 2002.

25. Bhaskaran K, Gasparrini A, Hajat S, et al. Time series regression studies in environmental epidemiology. Int J Epidemiol 2013; 42: 1187–1195.

For peer review only

26. Wahlberg AC, Wredling R. Telephone nursing: Calls and caller satisfaction. Int J Nurs Pract; 5: 164–170.

27. Hennekens CH, Buring JE, Mayrent SL. Epidemiology in Medicine. First edition. United States of America, 1987.

28. Krumpal I. Determinants of social desirability bias in sensitive surveys: a literature review. Qual Quant 2013; 47: 2025–2047.

29. Gorman E, Leyland AH, McCartney G, et al. Assessing the Representativeness of Population-Sampled Health Surveys Through Linkage to Administrative Data on Alcohol-Related Outcomes. Am J Epidemiol 2014; 180: 941–948.

30. Kaminsky E, Carlsson M, Röing M, et al. ‘If I didn’t trust Swedish Healthcare Direct, I would never call’ – views of making pediatric health calls. Clin Nurs Stud 2013; 1: 57.

31. Winneby E, Flensner G, Rudolfsson G. Feeling rejected or invited: Experiences of persons seeking care advice at the Swedish Healthcare Direct organization. Jpn J Nurs Sci 2014; 11: 87–93.

32. Bernal JL, Cummins S, Gasparrini A. Interrupted time series regression for the evaluation of public health interventions: a tutorial. Int J Epidemiol 2017; 46: 348–355.

33. Penfold RB, Zhang F. Use of Interrupted Time Series Analysis in Evaluating Health Care Quality Improvements. Acad Pediatr 2013; 13: S38–S44.

For peer review only

34. Wahlberg AC, Cedersund E, Wredling R. Bases for assessments made by telephone advice nurses. J Telemed Telecare 2005; 11: 403–407.

35. Bjorkman A, Engstrom M, Olsson A, et al. Identified obstacles and prerequisites in telenurses' work environment - a modified Delphi study. BMC Health Serv Res 2017; 17: 357.

36. Holmström I, Dall'Alba G. `Carer and gatekeeper' – conflicting demands in nurses' experiences of telephone advisory services. Scand J Caring Sci 2002; 16: 142–148.

37. Wahlberg AC, Cedersund E, Wredling R. Telephone nurses' experience of problems with telephone advice in Sweden. J Clin Nurs 2003; 12: 37–45.

38. Holmström I, Höglund AT. The faceless encounter: ethical dilemmas in telephone nursing. J Clin Nurs 2007; 16: 1865–1871.

39. Ernesäter A, Engström M, Holmström I, et al. Incident reporting in nurse-led national telephone triage in Sweden: the reported errors reveal a pattern that needs to be broken. J Telemed Telecare 2010; 16: 243–247.

40. Bibi M, Attwell RW, Fairhurst RJ, et al. The usage of NHS Direct by different ethnic and gender groups in an urban population. Rev Env Health 2008; 23: 235–41.

41. Shah SM, Cook DG. Socio-economic determinants of casualty and NHS Direct use. J Public Health Oxf 2008; 30: 75–81.

For peer review only

458 42. Cook EJ, Randhawa G, Large S, et al. A U.K. case study of who uses NHS direct: investigating the impact of age, gender, and deprivation on the utilization of NHS direct. Telemed J E Health 2012; 18: 693–8.

461 43. Cook EJ, Randhawa G, Large S, et al. Who uses NHS Direct? Investigating the impact of ethnicity on the uptake of telephone based healthcare. Int J Equity Health 2014; 13: 99.

464 44. Rysst Gustafsson S, Eriksson I. Quality indicators in telephone nursing–An integrative review. Nursing Open. 2020. 00:1-13.

466

For peer review only

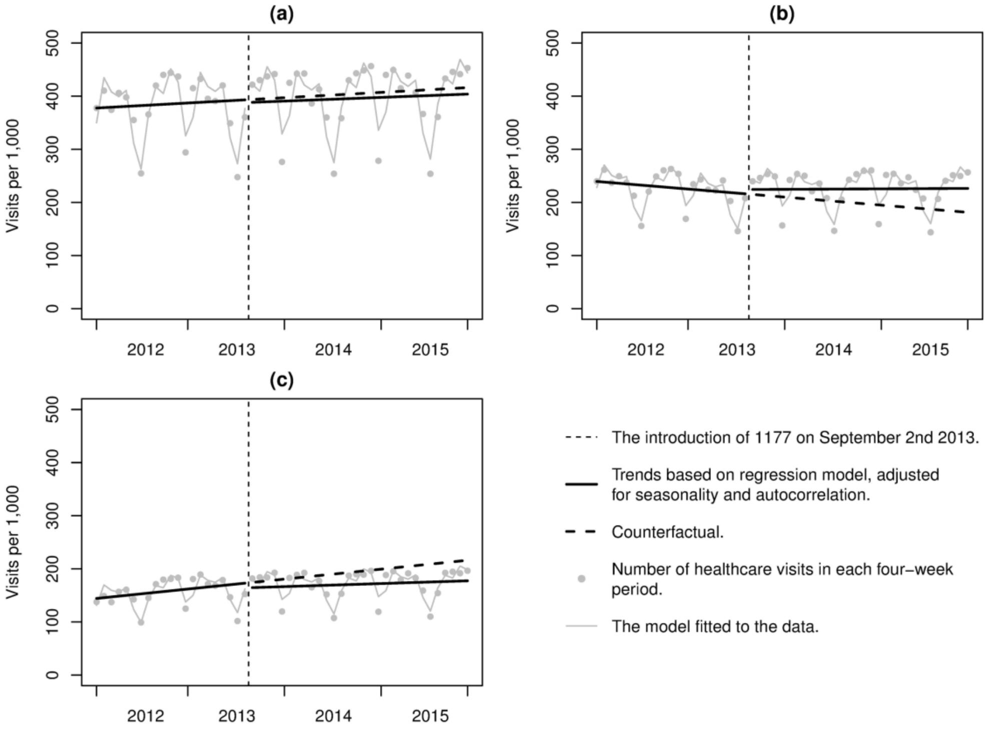

Figure 1

211x155mm (300 x 300 DPI)

STROBE 2007 (v4) Statement—Checklist of items that should be included in reports of *cohort studies*

Section/Topic	Item #	Recommendation	Reported on page #
**Title and abstract**	1	(*a*) Indicate the study's design with a commonly used term in the title or the abstract	1
		(*b*) Provide in the abstract an informative and balanced summary of what was done and what was found	3-4
**Introduction**			
Background/rationale	2	Explain the scientific background and rationale for the investigation being reported	6-7
Objectives	3	State specific objectives, including any prespecified hypotheses	7
**Methods**			
Study design	4	Present key elements of study design early in the paper	8
Setting	5	Describe the setting, locations, and relevant dates, including periods of recruitment, exposure, follow-up, and data collection	8-11
Participants	6	(*a*) Give the eligibility criteria, and the sources and methods of selection of participants. Describe methods of follow-up	9-11
		(*b*) For matched studies, give matching criteria and number of exposed and unexposed	n.a.
Variables	7	Clearly define all outcomes, exposures, predictors, potential confounders, and effect modifiers. Give diagnostic criteria, if applicable	9-11
Data sources/ measurement	8*	For each variable of interest, give sources of data and details of methods of assessment (measurement). Describe comparability of assessment methods if there is more than one group	*9-11*
Bias	9	Describe any efforts to address potential sources of bias	11
Study size	10	Explain how the study size was arrived at	n.a.
Quantitative variables	11	Explain how quantitative variables were handled in the analyses. If applicable, describe which groupings were chosen and why	10-11
Statistical methods	12	(*a*) Describe all statistical methods, including those used to control for confounding	10-11
		(*b*) Describe any methods used to examine subgroups and interactions	10-11
		(*c*) Explain how missing data were addressed	9
		(*d*) If applicable, explain how loss to follow-up was addressed	n.a.
		(*e*) Describe any sensitivity analyses	n.a.
**Results**			

For peer review only

Participants	13*	(a) Report numbers of individuals at each stage of study—eg numbers potentially eligible, examined for eligibility, confirmed eligible, included in the study, completing follow-up, and analysed	12
		(b) Give reasons for non-participation at each stage	n.a.
		(c) Consider use of a flow diagram	n.a.
Descriptive data	14*	(a) Give characteristics of study participants (eg demographic, clinical, social) and information on exposures and potential confounders	12
		(b) Indicate number of participants with missing data for each variable of interest	9
		(c) Summarise follow-up time (eg, average and total amount)	12
Outcome data	15*	Report numbers of outcome events or summary measures over time	12-13
Main results	16	(*a*) Give unadjusted estimates and, if applicable, confounder-adjusted estimates and their precision (eg, 95% confidence interval). Make clear which confounders were adjusted for and why they were included	n.a.
		(*b*) Report category boundaries when continuous variables were categorized	12-13
		(*c*) If relevant, consider translating estimates of relative risk into absolute risk for a meaningful time period	n.a.
Other analyses	17	Report other analyses done—eg analyses of subgroups and interactions, and sensitivity analyses	12-14
**Discussion**			
Key results	18	Summarise key results with reference to study objectives	15
**Limitations**			
Interpretation	20	Give a cautious overall interpretation of results considering objectives, limitations, multiplicity of analyses, results from similar studies, and other relevant evidence	18-19
Generalisability	21	Discuss the generalisability (external validity) of the study results	20
**Other information**			
Funding	22	Give the source of funding and the role of the funders for the present study and, if applicable, for the original study on which the present article is based	21-22

*Give information separately for cases and controls in case-control studies and, if applicable, for exposed and unexposed groups in cohort and cross-sectional studies.

Note: An Explanation and Elaboration article discusses each checklist item and gives methodological background and published examples of transparent reporting. The STROBE checklist is best used in conjunction with this article (freely available on the Web sites of PLoS Medicine at http://www.plosmedicine.org/, Annals of Internal Medicine at http://www.annals.org/, and Epidemiology at http://www.epidem.com/). Information on the STROBE Initiative is available at www.strobe-statement.org.

For peer review only

BMJ Open

An observational study of the implementation of telephone advice nursing in Sweden: did callers follow recommendations and did the rate of healthcare visits change?

Journal:	*BMJ Open*
Manuscript ID	bmjopen-2021-051233.R1
Article Type:	Original research
Date Submitted by the Author:	02-Jul-2021

Complete List of Authors:	Sundberg, Amanda; Karolinska Institute Department of Learning Informatics Management and Ethics<br>Wahlberg, Anna Carin; Karolinska Institutet Institutionen för neurobiologi vårdvetenskap och samhälle, Department of Neurobiologi<br>Zethraeus, N; Karolinska Institute Department of Learning Informatics Management and Ethics<br>Karampampa, Korinna; Karolinska Institute Division of Insurance Medicine, Department of Clinical Neuroscience

**Primary Subject Heading**:	Health economics
Secondary Subject Heading:	Nursing
Keywords:	Telemedicine < BIOTECHNOLOGY & BIOINFORMATICS, HEALTH SERVICES ADMINISTRATION & MANAGEMENT, PRIMARY CARE, HEALTH ECONOMICS, Organisation of health services < HEALTH SERVICES ADMINISTRATION & MANAGEMENT

SCHOLARONE™
Manuscripts

I, the Submitting Author has the right to grant and does grant on behalf of all authors of the Work (as defined in the below author licence), an exclusive licence and/or a non-exclusive licence for contributions from authors who are: i) UK Crown employees; ii) where BMJ has agreed a CC-BY licence shall apply, and/or iii) in accordance with the terms applicable for US Federal Government officers or employees acting as part of their official duties; on a worldwide, perpetual, irrevocable, royalty-free basis to BMJ Publishing Group Ltd ("BMJ") its licensees and where the relevant Journal is co-owned by BMJ to the co-owners of the Journal, to publish the Work in this journal and any other BMJ products and to exploit all rights, as set out in our licence.

The Submitting Author accepts and understands that any supply made under these terms is made by BMJ to the Submitting Author unless you are acting as an employee on behalf of your employer or a postgraduate student of an affiliated institution which is paying any applicable article publishing charge ("APC") for Open Access articles. Where the Submitting Author wishes to make the Work available on an Open Access basis (and intends to pay the relevant APC), the terms of reuse of such Open Access shall be governed by a Creative Commons licence – details of these licences and which Creative Commons licence will apply to this Work are set out in our licence referred to above.

Other than as permitted in any relevant BMJ Author's Self Archiving Policies, I confirm this Work has not been accepted for publication elsewhere, is not being considered for publication elsewhere and does not duplicate material already published. I confirm all authors consent to publication of this Work and authorise the granting of this licence.

For peer review only

Title:

An observational study of the implementation of telephone advice nursing in Sweden: did callers follow recommendations and did the rate of healthcare visits change?

Authors:

Amanda Sundberg[1], Anna Carin Wahlberg[2], Niklas Zethraeus[3], Korinna Karampampa[4]

Affiliations:

[1] Employee at Inera AB, Stockholm, Sweden, and Alumni of Master of Medical Science with a Major in Public Health Sciences from the Department of Learning, Informatics, Management and Ethics, Karolinska Institutet, Stockholm, Sweden

[2] Division of Nursing, Department of Neurobiology, Care Sciences and Society, Karolinska Institutet, Stockholm, Sweden

[3] Department of Learning, Informatics, Management and Ethics, Karolinska Institutet

[4] Division of Insurance Medicine, Department of Clinical Neuroscience, Karolinska Institutet, Stockholm, Sweden

For peer review only

Corresponding author:

Korinna Karampampa

Division of Insurance Medicine,

Department of Clinical Neuroscience,

Karolinska Institutet, Stockholm, Sweden

Email: Korinna.karampampa@ki.se

For peer review only

Abstract

Objectives: Telephone advice nursing is introduced in many countries with one key aim being a reduction of avoidable healthcare visits. The aim of this study was to explore whether callers to a telephone advice nursing service followed the telenurses' recommendations, and whether there was a change in the level and trend of the rate of healthcare visits after the introduction of telephone advice nursing.

Design: Observational study.

Setting: Primary and secondary care in Jönköping Region, Sweden.

Participants: Telephone advice nursing calls, 06-23 hours, 2014-2015 (n=185,994) and outpatient healthcare visits 2012-2015 (n=6,877,266).

Primary outcome: Proportion of callers who visited healthcare within the time period advised by the telenurse.

Secondary outcome: Change in level or trend of the overall rate of healthcare visits per 1,000 persons and four-week period after the introduction of telephone advice nursing, with subgroup analysis for primary and secondary care.

Results: 77 % of callers who were recommended either to visit healthcare within 24 hours or to 'wait and see' followed the recommendations. There was no significant change in level (-5.15; 95% CI -15.80 to 5.50; *p*=0.349) or trend (-0.24; 95% CI -0.86 to 0.38; *p*=0.448) of the overall rate of visits per 1,000 persons and four-week period after the introduction of telephone advice nursing. For the rate of primary care visits, an increase in level (8.01; 95% CI 6.36 to 9.66; *p*<0.001) and trend (1.28; 95%

For peer review only

CI 1.17 to 1.39*; p*<0.001) were observed. For the rate of secondary care visits, a decrease in level (-8.77, 95% CI -14.41 to -3.13; *p*=0.004) and trend (-1.03, 95% CI -1.35 to -0.71; *p*<0.001) were observed.

Conclusions: The introduction of telephone advice nursing may have contributed to a shift in the rate of healthcare visits from secondary to primary care.

Keywords: Telephone advice nursing, Telenursing, telephone advice nursing, Telehealth, Interrupted time series analysis

Strengths and limitations of this study

- *Observational study based on registry data covering four years and over 7 million observations.*
- *Statistical analysis using interrupted time series analysis with segmented regression, a recommended research design when randomisation is not possible.*
- *No control series was used to control for potential confounders in the interrupted time series analysis.*
- *Results might not be generalizable to the rest of Sweden, or to other countries.*

For peer review only

Background

A striking development around the Western world is the increased availability of telephone triage and advice services (henceforth telephone advice nursing), where telephone nurses (henceforth telenurses) provide callers with triage, advice, support, education, referral to adequate care levels, healthcare information and coordinate healthcare resources.[1] Telephone advice nursing has been implemented in the United Kingdom, Denmark, Sweden, Australia, the USA, the Netherlands and Canada.[2–4] In Sweden, the telephone advice nursing service 1177 Vårdguiden (henceforth 1177) was implemented successively across the regions between 2003-2013.[5] The service has the five minimum components required to ensure patient safety: qualified staff, medically approved guidelines, electronic medical records, staff training programmes and standards.[6,7]

One key aim from policymakers when implementing telephone advice nursing services is helping care seekers find the appropriate level of care and reducing unnecessary healthcare visits.[8,4,9,10] Swedish healthcare providers hypothesised that 1177 would contribute to a more efficient healthcare system.[11–13] It was predicted that if callers followed the telenurses' recommendations, the service would impact upon the rates of healthcare visits as a greater proportion of patients would be able to self-care or seek care at more appropriate healthcare levels than they would without access to telephone advice nursing.[11] The anticipated effect from this transition was an overall reduction of costly emergency visits to secondary care, which would free resources in the healthcare system. The rate of primary care visits was expected to remain more or less unchanged but with a more appropriate case mix.[11]

For peer review only

The available evidence worldwide regarding the extent to which callers follow recommendations from telenurses and telephone advice nursing's impact on healthcare visits is inconsistent.[2,4,8,10] Context-specific factors such as healthcare organization and workplace environment affect the external validity of findings.[8,14] Identifying key policy lessons for any given telephone advice nursing service therefore requires it to be studied specifically.

For 1177, evidence regarding the extent to which callers follow recommendations from telenurses is diverse. 1177's own surveys to callers find that 90 % follow the recommendations,[15] while a government agency used observational data to find that 55 – 78 % follow recommendations depending on the advice provided.[16]

Similarly, evidence regarding 1177's impact on the rate of healthcare visits is scarce. Four studies have found telephone advice nursing services in Sweden to redistribute visits to less costly levels of care.[17–20] Two of the studies concerned local predecessors to 1177.[17,18] All studies based their conclusions on self-reported survey data rather than observational data, which possibly limits the strength of the evidence.

To explore the hypothesis that 1177 may have freed resources in the healthcare system through reducing avoidable healthcare visits, the aim of this study was to use observational data to evaluate whether 1177 callers follow the telenurses' recommendations, and whether there was any change in the level and trend of healthcare visits after the introduction of telephone advice nursing.

For peer review only

Methods

Setting

This observational study used patient-level data from the patient administrative system in Jönköping Region, Sweden. The region manages a tax-financed healthcare system with universal coverage for its 345,000 inhabitants.[21–24] 1177 was made available to all inhabitants in the region on September 2nd 2013. Before that, telephone advice nursing was provided by individual hospitals and health care centres in the region. When 1177 was introduced, seven regional telephone advice lines were closed, including those to emergency departments and out-of-hours centres. No other large changes to healthcare in the region were carried out in 2012-2015.[21–24]

In contrast to previous advice lines, 1177 was always open and staffed with nurses specifically trained in telephone advice nursing.[7] The service followed national standards in telephone advice nursing, with a key component being consistent use of the national symptom-based computer decision support system (CDSS). The CDSS contained symptoms with related urgency levels, as well as a referral catalogue of available health care services with opening times, addresses as well as any specific rules about which patient groups different healthcare units receive. The nurses identified the callers' main symtpom and thereby obtained the urgency level from the CDSS. They thereafter used the catalogue in combination with their knowledge of the healthcare organization to find care that would meet the callers' needs within the urgency level, given factors such as opening times, geography and accessibility. The region intended 1177 to be a first point of contact with healthcare for inhabitants, along with the options

For peer review only

of reading information on the 1177 website, calling a healthcare centre or visiting an emergency department or out-of-hours care centre.[25]

Study cohort

To determine the percentage of callers who follow the recommendations of the telenurse, calls to 1177 were linked to information on any subsequent outpatient healthcare visits (emergency visits, visits that required referrals and visits that did not require referrals). The linkage and subsequent anonymization were performed by the system administrator using the unique personal identity number that Swedish residents have. Only anonymized patient-level data was used in the study.

To examine whether there was any change in the level and trend of healthcare visits after 1177 was introduced, anonymized patient-level data on all outpatient healthcare visits in the region (emergency visits, visits that required referrals and visits that did not require referrals) was used.

The study was approved by the Swedish Ethical Review Authority (2019-01784).

Definitions and statistical analysis

The first study outcome of whether callers followed recommendations of the telenurse was defined as the proportion of callers who visited healthcare within the time period advised by the telenurse.

To evaluate this outcome, calls to 1177 between 6 am and 11 pm and any subsequent healthcare visit at any time of day, that took place within 72 hours of the call between January 1st 2014 and December 31st 2015 (n = 185,994) were used. Calls that referred

For peer review only

patients to healthcare outside of the region were excluded (n = 1,839). Night-time calls between 11 pm and 6 am were unavailable for study as they were answered by a neighbouring county council on behalf of Jönköping Region.

Variables included date and time of call, callers' age and sex, the 'urgency level', as well as the name of any healthcare unit visited within 72 hours of the call, and if the visit occurred within 24 hours.

The variable 'urgency level' described how urgent the telenurse found it for the caller to visit healthcare. The variable had five different levels: 'immediately', 'quickly' (meaning 'within a few hours'), 'within 24 hours', 'a weekday in near time' (meaning within a week or a few weeks) and 'wait and see' (meaning no need to visit healthcare). These five levels were collapsed into three levels, since the data did not allow following the caller's subsequent behaviour on a more granular level:

- Visit within 24 hours (including 'immediately', 'quickly' and 'within 24 hours')
- Visit within a few weeks (including 'a weekday in near time')
- 'Wait and see' (including 'wait and see')

Each call was mapped to the data on healthcare visits, providing the proportion of callers that were recommended to visit within 24 hours and did so, as well as the proportion of callers recommended to 'wait and see' that did not visit healthcare within 72 hours of the call. The proportion of callers who followed the recommendation to visit healthcare within a few weeks could not be determined as the data on healthcare visits only stretched to 72 hours after the call.

For peer review only

To evaluate the second study outcome, i.e. to determine whether the rate of healthcare visits changed after the introduction of 1177, all outpatient healthcare visits between January 23, 2012 and December 20, 2015 were used (n=6,877,266). Visits made by patients not registered as residents in the region were excluded (n=215,471). Interrupted time series with segmented regression was used to study this outcome, because it is a robust research design where randomised controlled trials are not possible.[26–29] The central assumption of the method was that the trend of healthcare visits before the introduction of telephone advice nursing would have continued unchanged if the service had not been introduced.

The data covered 51 four-week periods; 21 before 1177 was implemented and 30 after. Variables included date and time of the visit. The series' data points were calculated as visits per 1,000 population and four-week period. The rates were controlled for population growth by using the population size for each given year as the denominator.

Three time series were made, one for all outpatient visits (primary and secondary care) and one for each subgroup of outpatient primary and secondary care visits. To create the subgroups for outpatient primary and secondary care, the healthcare units were categorised according to a definition from the Swedish Agency for Health and Care Services:[16]

- Primary care: primary care centres, physicians' centres, out-of-hours primary care, children's healthcare centres and maternal healthcare centres.
- Secondary care: specific or hospital-based specialist care including emergency care.

For peer review only

Regression models with two segments were fitted to the data, one for the period pre-1177 and one for the period after. Separate levels (intercepts) and trends (slopes) were estimated for each segment.[27] Statistical tests were carried out to estimate changes in the level or trend after the introduction of 1177. To estimate the size of the effect, the models were used to calculate the rates one year (12 four-week periods) after 1177 was introduced and the relative changes compared to the rates that would have been if the trend before 1177 was introduced had continued unchanged.

To control for seasonality, harmonic sine and cosine pairs (‘Fourier terms’) were added to the model.[30] To control for autocorrelation, a moving averages term of order q=13 was added using generalized least squares regression with a maximum likelihood method. Three plausible alternative autocorrelation terms were tested using likelihood-ratio tests. Since the Akaike information criterion did not indicate improvements to the fit of the model, q=13 was retained. Nevertheless, some autocorrelation remained.

Patient and public involvement

Patients and public were not involved in the design and conduct of this study.

For peer review only

Results

Calls to the telephone advice nursing service represented 5 % of outpatient contacts. The proportion that followed the recommendation differed between groups (Table 1). A majority of those recommended to visit healthcare within 24 hours did so (73 %), and 84 % of those recommended to 'wait and see' did not visit healthcare within 72 hours. Combining these groups, 77 % followed the recommendation. When stratified by age groups and sex, the proportion that followed the telenurses' recommendation varied between 66 % (females aged 20-39) and 90 % (children aged 0-9).

Table 1. Callers that followed the recommendation to visit healthcare or 'wait and see', Jönköping Region 2014-2015.

		Followed the recommendation to visit healthcare within 24 h n(%)		Followed the recommendation to 'wait and see' n(%)		Both groups combined n(%)	
	All (n=162,614)	75,159	(73)	50,836	(84)	125,995	(77)
	Males (n=72,180)	34,403	(75)	22,533	(86)	56,936	(79)
	Females (n=90,434)	40,756	(72)	28,303	(83)	69,059	(76)
All	Age group	Followed the recommendation to visit healthcare within 24 h n(%)		Followed the recommend-action to 'wait and see' n(%)		Both groups combined n(%)	
	0-9 (n=51,412)	18,925	(70)	22,045	(90)	40,970	(80)
	10-19 (n=16,032)	8,559	(79)	4,388	(84)	12,947	(81)
	20-39 (n=43,489)	18,605	(68)	13,273	(82)	31,878	(73)
	40-59 (n=25,289)	13,972	(77)	5,624	(80)	19,596	(77)
	>60 (n=26,392)	15,098	(79)	5,505	(77)	20,603	(78)
Males	Age group						
	0-9 (n=27,562)	10,246	(71)	11,769	(90)	22,015	(80)
	10-19 (n=7,365)	4,065	(81)	2,857	(84)	6,044	(82)
	20-39 (n=15,488)	7,232	(71)	4,496	(84)	11,728	(76)
	40-59 (n=10,706)	6,223	(78)	2,207	(80)	8,430	(79)
	>60 (n=11,059)	6,637	(80)	2,082	(76)	8,719	(79)
Females	Age group						
	0-9 (n=23,850)	8,679	(70)	10,276	(90)	18,955	(79)
	10-19 (n=8,667)	4,494	(78)	2,409	(83)	6,903	(80)
	20-39 (n=28,001)	11,373	(66)	8,777	(81)	20,150	(72)
	40-59 (n=14,583)	7,749	(76)	3,417	(79)	11,166	(77)
	>60 (n=15,333)	8,461	(78)	3,423	(77)	11,884	(78)

For peer review only

For peer review only - http://bmjopen.bmj.com/site/about/guidelines.xhtml

Overall, for all healthcare visits, the final segmented regression model showed no significant changes in level (-5.15; 95% CI -15.8 to 5.5; *p*=0.349) or trend (-0.24; 95% CI -0.86 to 0.38; *p*=0.448) of the healthcare visits after the introduction of 1177. Statistically significant changes after the introduction of 1177 were found only in the subgroup analysis. Immediately after the introduction of 1177, primary care visits had an increase of 8.01 visits per 1,000 persons (95% CI 6.36 to 9.66; *p*<0.001) and had a trend increase of 1.28 visits per 1,000 per four-week period (95% CI 1.17 to 1.39; *p*<0.001). In contrast, visits to secondary care decreased with 8.77 visits per 1,000 weeks immediately after the introduction of telephone advice nursing (95% CI -14.41 to -3.13; *p*=0.004), and had a trend decrease of 1.03 visits per 1,000 per four week period (% CI -1.35 to -0.71; *p*<0.001) (Table 2 and Figure 1).

When the regression models were used to estimate the effect size one year (12 four-week periods) after the introduction of 1177, an 11.5 % increase in the rate of primary care visits and an 11 % decrease in the rate of secondary care visits was found (Table 3).

Table 2. Results of the interrupted time series analysis for healthcare visits, Jönköping Region 2012-2015.

	Level (intercept)	Trend (slope)	Change in level after telephone advice nursing	Change in trend after telephone advice nursing
All visits (primary and secondary care)				
Co-efficient	376.909	0.769	-5.152	-0.244
95% CI	368.501 to 385.317	0.058 to 1.480	-15.797 to 5.497	-0.863 to 0.383
p-value	<0.001	0.040	0.349	0.448
Primary care visits				
Co-efficient	240.49	-1.164	8.013	1.279
95% CI	238.738 to 242.242	-1.297 to -1.031	6.36 to 9.66	1.17 to 1.39
p-value	<0.001	<0.001	<0.001	<0.001
Secondary care visits				

For peer review only - http://bmjopen.bmj.com/site/about/guidelines.xhtml

For peer review only

Co-efficient	142.861	1.437	-8.767	-1.031
95% CI	138.594 to 147.128	1.074 to 1.8	-14.413 to -3.127	-1.349 to -0.711
p-value	<0.001	<0.001	0.004	<0.001

Figure 1. Interrupted time series analysis for the rate of healthcare visits, Jönköping Region 2012-2015. (a) All visits (primary and secondary care). (b) Primary care visits. (c) Secondary care visits. [1]

-------------Insert Figure 1-------------

Table 3. Observed rates of healthcare visits 12 four-week periods after telephone advice nursing was introduced and rates if the trends observed pre-implementation would have continued unchanged.

	All visits (primary and secondary care) (p=0.448)	Primary care visits (p<0.001)	Secondary care visits (p<0.001
Observed rate per 1,000 persons	394.20	225.59	169.13
Rate per 1,000 persons if the trend observed pre-implementation would have continued unchanged	402.28	202.23	190.27
Absolute change (observed rate– rate if the trend observed pre-implementation would have continued unchanged)	-8.02	23.36	-21.14
Relative change	-2.01 %	11.55 %	-11.11%

For peer review only

[1] In these graphical displays of the interrupted time series analyses, the trends of the rates of health care visits pre- and post- implementation of 1177 are shown (the thick solid lines). The pre-implementation trend has been extrapolated to the post-implementation period (the thick dotted line), to show what would have happened if the pre-implementation trends would have continued unchanged. The data points used for the analysis are shown in grey. The model fitted to the data show the seasonal trends (the thin grey lines).

Discussion

To summarize, the findings of this study indicate that large proportions (77%) of those who call 1177 follow the recommendations, and that there was in a shift in healthcare visits from secondary to primary care after the introduction of 1177. It is possible that the introduction of 1177 contributed to these changes, enabling more patients to seek care at lower levels than they could prior to the service's introduction.

The observed changes in the rate of healthcare visits broadly support the findings of previous survey-based studies in Sweden, which have suggested that telephone advice nursing services in the Swedish setting reduce emergency visits.[17–19] The interpretation that 1177 contributed to the observed changes in the rates of healthcare visits is also strengthened by the lack of plausible alternative explanations: the supply of primary and secondary care was stable throughout the study period and no other potential confounders were found.[21–24]

Compared to 1177's own survey-based findings on the proportions that follow the recommendations of the telenurse (90 %), 77 % is lower.[15] This is also the case when considering earlier survey-based studies on local predecessors to 1177.[1,17,18,31] However, the finding is similar to those of a government agency report that used observational data.[16] This contrast between survey data and observational data is supported by systematic reviews of the international literature on telephone advice nursing, according to which self-reported data tends to provide higher proportions than observational data.[2]

For peer review only

For peer review only - http://bmjopen.bmj.com/site/about/guidelines.xhtml

These inconsistent results of survey and observational data respectively may be considered as complementary as they to some extent measure different constructs. While observational studies require researchers to define what it means to follow the recommendations of the telenurse, survey-based studies let respondents interpret its meaning subjectively.[32] Although the latter study design suffers from threats to validity, primarily from recall-, social desirability- and nonresponse bias,[33–35] it also allows for capturing nuances of what it means to follow recommendations, and for studying the patient's perspective.

There are a broad range of possible care-seeking scenarios concealed behind the proportions found in this study. First, when callers appear to have followed the recommendations, it is not certain that the call to 1177 were what led them to do what they did. They may have based their decisions on consultations with primary care, family and friends, websites, and so forth. Second, the data in this study only showed the telenurse's main recommendation and cannot reflect potential alternative courses of action that the telenurse and caller may have also discussed, for example depending on how the patient's health status developed. Third, care sought after a call to 1177 within the time recommended by the nurse may have been for a separate issue than that discussed with the telenurse. Fourth, care recommended by the telenurse may have been inaccessible to the caller, making it impossible to follow the recommendations. Finally, callers may have chosen not to follow the recommendations because they lacked confidence in the telenurse or found the recommendations inappropriate.[20,36,37]

For peer review only

Strengths and limitations of the study

The strength of the study is that complete anonymized patient-level data was used, providing a strong design for answering the research questions. Unlike previous studies, this study's findings were derived from a large number of observations, free from the biases of surveys.[33] The interrupted time series analysis was the next-best method for studying the rate of healthcare visits before and after the introduction of 1177 given a randomised, controlled trial was not possible.[27–29]

This study also has several limitations. Ideally, a control series should have been used to control for potential confounders.[27] In lack of a control group, media archives and grey literature were searched to identify potential confounding events, but none were found.[21–24]

The autocorrelation that remained in the segmented regression model fitted to the data, despite attempts to control for it, is another limitation. Autocorrelation does not affect coefficients, but it does affect the standard errors, which means we cannot rely on the p-values. This is particularly the case when p-values are close to 0.05. In the results of this study, however, p-values for most statistically significant results were <0.001.

Finally, a limitation concerns the generalizability of the results. First, this study has focused on 1177 in one Swedish region. While the healthcare organization across the regions in Sweden do not differ substantially, some differences may be present regarding the availability of healthcare services and the population demographics. Therefore, the results might not be generalizable to the rest of Sweden, or to other countries.

For peer review only

For peer review only - http://bmjopen.bmj.com/site/about/guidelines.xhtml

In addition, due to the unavailability of data, night calls during the hours 11pm and 6am were excluded from this study. However, it is unlikely that the behaviour of night-time callers would be substantially different than that of people calling during the rest of the day. Still, since the level of urgency may be different between e.g. morning or night calls, the results of this study may be not generalizable to calls/callers throughout the entire day.

Implications for future research and policy

For policy makers, this study provides some support that the implementation of telephone advice nursing contributed to their aims of freeing resources in the healthcare system, as a large proportion of callers followed the nurses' recommendations, and as the service may have contributed to the change in the rate of healthcare visits. The implication for policy is that telephone advice nursing might be considered as a positive element in the healthcare organisation.

Three areas should be of specific interest to future research and policy. One is the effects of technology used in telephone advice nursing: given the rapid technological developments such as automatic triage and the use of photos and video calls in telephone advice nursing, it should be of interest to find out more about how technology is being used and can be used to develop and enhance telephone advice nursing and its role in the healthcare system.[8]

Another area to further study is how the interaction between telephone advice nursing and other services in the healthcare system affect efficiency. For example, studies have established that one of the largest challenges experienced by telenurses at 1177 in

For peer review only

For peer review only - http://bmjopen.bmj.com/site/about/guidelines.xhtml

Sweden is the lack of adequate resources in the healthcare organisation, with insufficient availability of primary care being the largest problem.[3,6,38–43] A recent study has also shown that the healthcare organisation is not adapted to meet the needs of callers with mental illness.[44] If the healthcare organisation is not set up to meet the needs of the population, 1177 cannot contribute to system efficiency with its full potential. Future studies of 1177's contribution to system efficiency should treat it not as an isolated service but as one whose efficacy is interlinked with the functioning of the healthcare system as a whole.[45]

In addition, future research may focus on how access to, and use of, telephone advice nursing varies in the population. This study found that only 5 % of outpatient healthcare contacts were calls to 1177, raising the question of whether more patients could benefit from using the service. This aspect has not been studied in Sweden, but studies in the United Kingdom have found that the former telephone advice nursing service National Health Service Direct was used more by some groups. Important determinants were ethnicity, gender, age and socio-economic status.[46–49] If telephone advice nursing can contribute to inhabitants receiving care at the most appropriate levels, it is important that all groups gain equal access to the service.

For peer review only

Conclusions

The results indicate that large proportions of those who call 1177 follow the recommendations and that the introduction of 1177 in Region Jönköping may have contributed to a shift in healthcare visits from secondary to primary care. The results provide some support to the hypothesis that 1177 may have contributed to system efficiency, freeing resources through reducing unnecessary costly healthcare visits.

For peer review only - http://bmjopen.bmj.com/site/about/guidelines.xhtml

Declarations

Ethics approval and consent to participate

Informed consent was not collected from the participants of this study. The study was approved by the Swedish Ethical Review Authority, registered under number 2019-01784.

Availability of data and materials

The data that support the findings of this study are available from Region Jönköping, but restrictions apply to the availability of these data, which were used under license for the current study, and so are not publicly available.

Competing interests

AS is employed by Inera AB, a limited company owned by Swedish regions, municipalities as well as the Swedish Association of Local Authorities and Regions (SALAR) that administrates the technical solutions used in 1177. AS conducted this research as part of her master's degree project in the Master's programme in Public Health Sciences (track Health Economics, Policy and Management) at the Karolinska Institutet, while she was on leave of absence from her employment. The study findings have been interpreted scientifically by all co-authors, without the involvement of Inera AB, and not in the interest of 1177.

All other co-authors (ACW, NZ, and KK) are employed at or affiliated with Karolinska Institutet, Stockholm, Sweden.

For peer review only

KK is currently employed by BMS, a pharmaceutical company which is not involved with 1177 in Sweden.

Funding

This research received no specific grant from any funding agency in the public, commercial or not-for-profit sectors.

Authors' Contributions

AS, NZ and KK contributed to the conceptualization of the research questions, study design and methods. AS performed the analysis of the data, interpreted the study findings, and drafted the manuscript. All remaining authors, AS, NZ, KK and ACW assisted in the interpretation of the study findings and contributed to the manuscript with comments, suggestions and text.

Acknowledgements

Not applicable.

For peer review only

References

1 Wahlberg AC. Telephone advice nursing: callers' perceptions, nurses' experience of problems and basis for assessments. 2004.

2 Lake R, Georgiou A, Li J, et al. The quality, safety and governance of telephone triage and advice services – an overview of evidence from systematic reviews. BMC Health Serv Res 2017;**17**. doi:10.1186/s12913-017-2564-x

3 Wahlberg AC, Bjorkman A. Expert in nursing care but sometimes disrespected-Telenurses' reflections on their work environment and nursing care. J Clin Nurs 2018;**27**:4203–11. doi:10.1111/jocn.14622

4 Gidora H, Borycki EM, Kushniruk AW. Effects of Telenursing Triage and Advice on Healthcare Costs and Resource Use. Stud Health Technol Inform 2019;**257**:133–9.

5 Leppänen V. Inledning. In: Telefonrådgivning inom hälso- och sjukvård. Hungary: : Studentlitteratur 2008.

6 Wheeler SQ, Greenberg ME, Mahlmeister L, et al. Safety of Clinical and Non-Clinical Decision Makers in Telephone Triage: A Narrative Review. J Telemed Telecare 2015;**21**:305–22. doi:10.1177/1357633X15571650

7 Inera. 1177 Vårdguiden på telefon - Inera. 2021.https://www.inera.se/tjanster/1177-vardguiden/1177-vardguiden-pa-telefon/ (accessed 24 Jun 2021).

8 Boggan JC, Shoup JP, Whited JD, et al. Effectiveness of Acute Care Remote Triage Systems: a Systematic Review. J Gen Intern Med 2020;**35**:2136–45. doi:10.1007/s11606-019-05585-4

9 Campbell JL, Fletcher E, Britten N, et al. Telephone triage for management of same-day consultation requests in general practice (the ESTEEM trial): a cluster-randomised controlled trial and cost-consequence analysis. Lancet 2014;**384**:1859–68. doi:10.1016/S0140-6736(14)61058-8

10 Ismail SA, Gibbons DC, Gnani S. Reducing inappropriate accident and emergency department attendances: a systematic review of primary care service interventions. Br J Gen Pr 2013;**63**:e813-20. doi:10.3399/bjgp13X675395

11 Landstingsförbundet. Vårdråd direkt - Sjukvårdsrådgivningar i samverkan. Slutrapport från utredningen om nationellt samordnad sjukvårdsrådgivning. Stockholm: : Landstingsförbundet 2003.

12 Stienstedt G. Effektiv vård: slutbetänkande av en nationell samordnare för effektivare resursutnyttjande inom hälso- och sjukvården. Stockholm: : Statens Offentliga Utredningar 2016.

For peer review only

13 Kaminsky E, Röing M, Björkman A, et al. Telephone nursing in Sweden: A narrative literature review. Nurs Health Sci 2017;**19**:278–86. doi:10.1111/nhs.12349

14 Lewinski AA, Rushton S, Van Voorhees E, et al. Implementing remote triage in large health systems: A qualitative evidence synthesis. Res Nurs Health 2021;**44**:138–54. doi:10.1002/nur.22093

15 Eklöf P. 1177 Vårdguiden på telefon. 2017.https://www.1177.se/Om-1177/1177-sjukvardsradgivning/ (accessed 12 Jan 2018).

16 En primär angelägenhet - kunskapsunderlag för en stärkt primärvård med patienten i centrum. Myndigheten för vård- och omsorgsanalys 2017.

17 Nordling S. Ekonomisk utvärdering av sjukvårdsupplysning per telefon i Jämtland och Malmö. Lund: : IHE 2001.

18 Marklund B, Ström M, Månsson J, et al. Computer-supported telephone nurse triage: an evaluation of medical quality and costs. J Nurs Manag 2007;**15**:180–7. doi:10.1111/j.1365-2834.2007.00659.x

19 Rahmqvist M, Husberg M. Effekter av sjukvårdsrådgivning per telefon - en analys av rådgivningsverksamheten 1177 i Östergötland och Jämtland. Linköping: : Linköpings universitet, Centrum för utvärdering av medicinsk teknologi 2009.

20 Martinsson J, Gustafsson S. Modeling the effects of telephone nursing on healthcare utilization. Int J Med Inf 2018;**113**:98–105. doi:10.1016/j.ijmedinf.2018.02.004

21 Region Jönköpings län. Årsredovisning 2015: med förvaltningsberättelse. 2016.

22 Region Jönköpings län. Årsredovisning 2014: med förvaltningsberättelse. 2015.

23 Landstinget i Jönköpings län. Årsredovisning 2012: med förvaltningsberättelse. Huskvarna: 2013.

24 Landstinget i Jönköpings län. Årsredovisning 2013: med förvaltningsberättelse. Ängelholm: 2014.

25 Landstingsstyrelsen, Landstinget i Jönköpings län. Införande av 1177 och ett gemensamt kundcenter. 2012.

26 Kontopantelis E, Doran T, Springate DA, et al. Regression based quasi-experimental approach when randomisation is not an option: interrupted time series analysis. BMJ 2015;**350**:h2750. doi:10.1136/bmj.h2750

27 Shadish WR, Cook TD, Campbell DT. Experimental and Quasi-Experimental Designs for Generalized Causal Inference. Belmont, California: : Wadsworth Cengage Learning 2002.

For peer review only

28 Bernal JL, Cummins S, Gasparrini A. Interrupted time series regression for the evaluation of public health interventions: a tutorial. Int J Epidemiol 2017;**46**:348–55. doi:10.1093/ije/dyw098

29 Penfold RB, Zhang F. Use of Interrupted Time Series Analysis in Evaluating Health Care Quality Improvements. Acad Pediatr 2013;**13**:S38–44. doi:10.1016/j.acap.2013.08.002

30 Bhaskaran K, Gasparrini A, Hajat S, et al. Time series regression studies in environmental epidemiology. Int J Epidemiol 2013;**42**:1187–95. doi:10.1093/ije/dyt092

31 Wahlberg AC, Wredling R. Telephone nursing: Calls and caller satisfaction. Int J Nurs Pract;**5**:164–70. doi:10.1046/j.1440-172x.1999.00179.x

32 Gustafsson S, Martinsson J, Wälivaara B-M, et al. Influence of self-care advice on patient satisfaction and healthcare utilization. J Adv Nurs 2016;**72**:1789–99. doi:10.1111/jan.12950

33 Hennekens CH, Buring JE, Mayrent SL. Epidemiology in Medicine. First edition. United States of America: 1987.

34 Krumpal I. Determinants of social desirability bias in sensitive surveys: a literature review. Qual Quant 2013;**47**:2025–47. doi:10.1007/s11135-011-9640-9

35 Gorman E, Leyland AH, McCartney G, et al. Assessing the Representativeness of Population-Sampled Health Surveys Through Linkage to Administrative Data on Alcohol-Related Outcomes. Am J Epidemiol 2014;**180**:941–8. doi:10.1093/aje/kwu207

36 Kaminsky E, Carlsson M, Röing M, et al. 'If I didn't trust Swedish Healthcare Direct, I would never call' – views of making pediatric health calls. Clin Nurs Stud 2013;**1**:57. doi:10.5430/cns.v1n3p57

37 Winneby E, Flensner G, Rudolfsson G. Feeling rejected or invited: Experiences of persons seeking care advice at the Swedish Healthcare Direct organization. Jpn J Nurs Sci 2014;**11**:87–93. doi:10.1111/jjns.12007

38 Wahlberg AC, Cedersund E, Wredling R. Bases for assessments made by telephone advice nurses. J Telemed Telecare 2005;**11**:403–7. doi:10.1177/1357633X0501100805

39 Bjorkman A, Engstrom M, Olsson A, et al. Identified obstacles and prerequisites in telenurses' work environment - a modified Delphi study. BMC Health Serv Res 2017;**17**:357. doi:10.1186/s12913-017-2296-y

40 Holmström I, Dall'Alba G. `Carer and gatekeeper' – conflicting demands in nurses' experiences of telephone advisory services. Scand J Caring Sci 2002;**16**:142–8. doi:10.1046/j.1471-6712.2002.00075.x

For peer review only

41 Wahlberg AC, Cedersund E, Wredling R. Telephone nurses' experience of problems with telephone advice in Sweden. J Clin Nurs 2003;**12**:37–45. doi:10.1046/j.1365-2702.2003.00702.x

42 Holmström I, Höglund AT. The faceless encounter: ethical dilemmas in telephone nursing. J Clin Nurs 2007;**16**:1865–71. doi:10.1111/j.1365-2702.2007.01839.x

43 Ernesäter A, Engström M, Holmström I, et al. Incident reporting in nurse-led national telephone triage in Sweden: the reported errors reveal a pattern that needs to be broken. J Telemed Telecare 2010;**16**:243–7. doi:10.1258/jtt.2009.090813

44 Bjorkman A, Salzmann-Erikson M. When all other doors are closed: Telenurses' experiences of encountering care seekers with mental illnesses. Int J Ment Health Nurs 2018;**27**:1392–400. doi:10.1111/inm.12438

45 Björkman A, Engström M, Winblad U, et al. Malpractice claimed calls within the Swedish Healthcare Direct: a descriptive - comparative case study. BMC Nurs 2021;**20**:21. doi:10.1186/s12912-021-00540-3

46 Bibi M, Attwell RW, Fairhurst RJ, et al. The usage of NHS Direct by different ethnic and gender groups in an urban population. Rev Env Health 2008;**23**:235–41.

47 Shah SM, Cook DG. Socio-economic determinants of casualty and NHS Direct use. J Public Health Oxf 2008;**30**:75–81. doi:10.1093/pubmed/fdn001

48 Cook EJ, Randhawa G, Large S, et al. A U.K. case study of who uses NHS direct: investigating the impact of age, gender, and deprivation on the utilization of NHS direct. Telemed J E Health 2012;**18**:693–8. doi:10.1089/tmj.2011.0256

49 Cook EJ, Randhawa G, Large S, et al. Who uses NHS Direct? Investigating the impact of ethnicity on the uptake of telephone based healthcare. Int J Equity Health 2014;**13**:99. doi:10.1186/s12939-014-0099-x

For peer review only

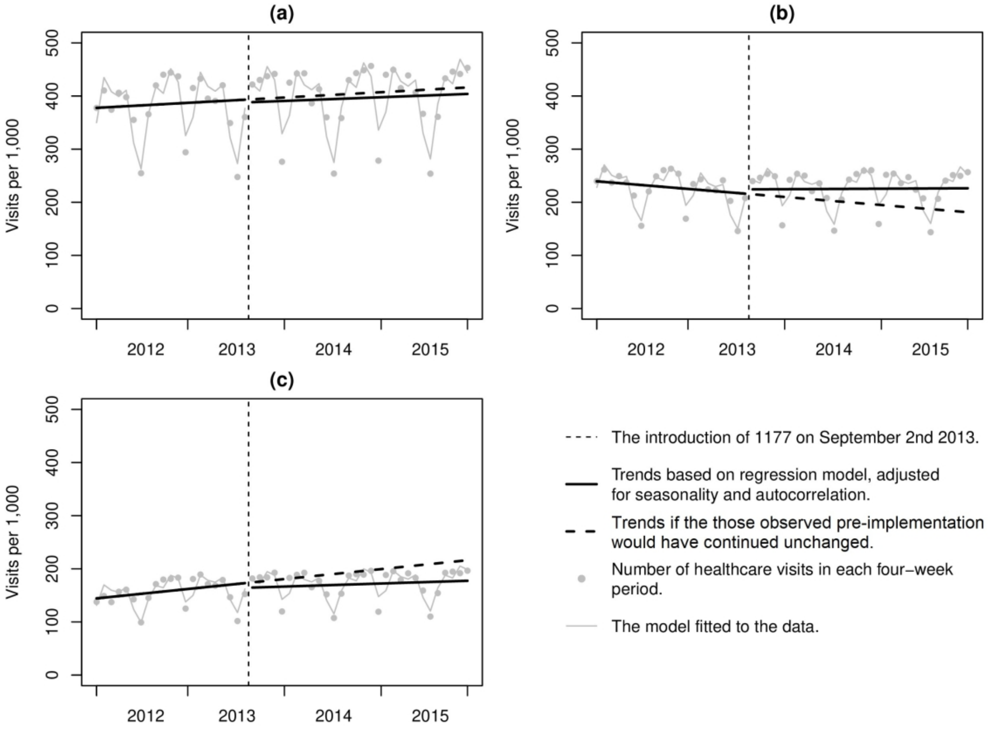

661x484mm (96 x 96 DPI)

STROBE 2007 (v4) Statement—Checklist of items that should be included in reports of *cohort studies*

Section/Topic	Item #	Recommendation	Reported on page #
**Title and abstract**	1	(*a*) Indicate the study's design with a commonly used term in the title or the abstract	1
		(*b*) Provide in the abstract an informative and balanced summary of what was done and what was found	3-4
**Introduction**			
Background/rationale	2	Explain the scientific background and rationale for the investigation being reported	6-7
Objectives	3	State specific objectives, including any prespecified hypotheses	7
**Methods**			
Study design	4	Present key elements of study design early in the paper	8
Setting	5	Describe the setting, locations, and relevant dates, including periods of recruitment, exposure, follow-up, and data collection	8-11
Participants	6	(*a*) Give the eligibility criteria, and the sources and methods of selection of participants. Describe methods of follow-up	9-11
		(*b*) For matched studies, give matching criteria and number of exposed and unexposed	n.a.
Variables	7	Clearly define all outcomes, exposures, predictors, potential confounders, and effect modifiers. Give diagnostic criteria, if applicable	9-11
Data sources/ measurement	8*	For each variable of interest, give sources of data and details of methods of assessment (measurement). Describe comparability of assessment methods if there is more than one group	*9-11*
Bias	9	Describe any efforts to address potential sources of bias	11
Study size	10	Explain how the study size was arrived at	n.a.
Quantitative variables	11	Explain how quantitative variables were handled in the analyses. If applicable, describe which groupings were chosen and why	10-11
Statistical methods	12	(*a*) Describe all statistical methods, including those used to control for confounding	10-11
		(*b*) Describe any methods used to examine subgroups and interactions	10-11
		(*c*) Explain how missing data were addressed	9
		(*d*) If applicable, explain how loss to follow-up was addressed	n.a.
		(*e*) Describe any sensitivity analyses	n.a.
**Results**			

For peer review only

Participants	13*	(a) Report numbers of individuals at each stage of study—eg numbers potentially eligible, examined for eligibility, confirmed eligible, included in the study, completing follow-up, and analysed	12
		(b) Give reasons for non-participation at each stage	n.a.
		(c) Consider use of a flow diagram	n.a.
Descriptive data	14*	(a) Give characteristics of study participants (eg demographic, clinical, social) and information on exposures and potential confounders	12
		(b) Indicate number of participants with missing data for each variable of interest	9
		(c) Summarise follow-up time (eg, average and total amount)	12
Outcome data	15*	Report numbers of outcome events or summary measures over time	12-13
Main results	16	(*a*) Give unadjusted estimates and, if applicable, confounder-adjusted estimates and their precision (eg, 95% confidence interval). Make clear which confounders were adjusted for and why they were included	n.a.
		(*b*) Report category boundaries when continuous variables were categorized	12-13
		(*c*) If relevant, consider translating estimates of relative risk into absolute risk for a meaningful time period	n.a.
Other analyses	17	Report other analyses done—eg analyses of subgroups and interactions, and sensitivity analyses	12-14
**Discussion**			
Key results	18	Summarise key results with reference to study objectives	15
**Limitations**			
Interpretation	20	Give a cautious overall interpretation of results considering objectives, limitations, multiplicity of analyses, results from similar studies, and other relevant evidence	18-19
Generalisability	21	Discuss the generalisability (external validity) of the study results	20
**Other information**			
Funding	22	Give the source of funding and the role of the funders for the present study and, if applicable, for the original study on which the present article is based	21-22

*Give information separately for cases and controls in case-control studies and, if applicable, for exposed and unexposed groups in cohort and cross-sectional studies.

Note: An Explanation and Elaboration article discusses each checklist item and gives methodological background and published examples of transparent reporting. The STROBE checklist is best used in conjunction with this article (freely available on the Web sites of PLoS Medicine at http://www.plosmedicine.org/, Annals of Internal Medicine at http://www.annals.org/, and Epidemiology at http://www.epidem.com/). Information on the STROBE Initiative is available at www.strobe-statement.org.

For peer review only

BMJ Open

An observational study of the implementation of telephone advice nursing in Sweden: did callers follow recommendations and did the rate of healthcare visits change?

Journal:	*BMJ Open*
Manuscript ID	bmjopen-2021-051233.R2
Article Type:	Original research
Date Submitted by the Author:	03-Aug-2021
Complete List of Authors:	Sundberg, Amanda; Karolinska Institute Department of Learning Informatics Management and Ethics<br>Wahlberg, Anna Carin; Karolinska Institutet Institutionen för neurobiologi vårdvetenskap och samhälle, Department of Neurobiologi<br>Zethraeus, N; Karolinska Institute Department of Learning Informatics Management and Ethics<br>Karampampa, Korinna; Karolinska Institute Division of Insurance Medicine, Department of Clinical Neuroscience
**Primary Subject Heading**:	Health economics
Secondary Subject Heading:	Nursing
Keywords:	Telemedicine < BIOTECHNOLOGY & BIOINFORMATICS, HEALTH SERVICES ADMINISTRATION & MANAGEMENT, PRIMARY CARE, HEALTH ECONOMICS, Organisation of health services < HEALTH SERVICES ADMINISTRATION & MANAGEMENT

SCHOLARONE™
Manuscripts

I, the Submitting Author has the right to grant and does grant on behalf of all authors of the Work (as defined in the below author licence), an exclusive licence and/or a non-exclusive licence for contributions from authors who are: i) UK Crown employees; ii) where BMJ has agreed a CC-BY licence shall apply, and/or iii) in accordance with the terms applicable for US Federal Government officers or employees acting as part of their official duties; on a worldwide, perpetual, irrevocable, royalty-free basis to BMJ Publishing Group Ltd ("BMJ") its licensees and where the relevant Journal is co-owned by BMJ to the co-owners of the Journal, to publish the Work in this journal and any other BMJ products and to exploit all rights, as set out in our licence.

The Submitting Author accepts and understands that any supply made under these terms is made by BMJ to the Submitting Author unless you are acting as an employee on behalf of your employer or a postgraduate student of an affiliated institution which is paying any applicable article publishing charge ("APC") for Open Access articles. Where the Submitting Author wishes to make the Work available on an Open Access basis (and intends to pay the relevant APC), the terms of reuse of such Open Access shall be governed by a Creative Commons licence – details of these licences and which Creative Commons licence will apply to this Work are set out in our licence referred to above.

Other than as permitted in any relevant BMJ Author's Self Archiving Policies, I confirm this Work has not been accepted for publication elsewhere, is not being considered for publication elsewhere and does not duplicate material already published. I confirm all authors consent to publication of this Work and authorise the granting of this licence.

For peer review only

Title:

An observational study of the implementation of telephone advice nursing in Sweden: did callers follow recommendations and did the rate of healthcare visits change?

Authors:

Amanda Sundberg[1], Anna Carin Wahlberg[2], Niklas Zethraeus[3], Korinna Karampampa[4]

Affiliations:

[1] Employee at Inera AB, Stockholm, Sweden, and Alumni of Master of Medical Science with a Major in Public Health Sciences from the Department of Learning, Informatics, Management and Ethics, Karolinska Institutet, Stockholm, Sweden

[2] Division of Nursing, Department of Neurobiology, Care Sciences and Society, Karolinska Institutet, Stockholm, Sweden

[3] Department of Learning, Informatics, Management and Ethics, Karolinska Institutet

[4] Division of Insurance Medicine, Department of Clinical Neuroscience, Karolinska Institutet, Stockholm, Sweden

For peer review only

Corresponding author:

Korinna Karampampa

Division of Insurance Medicine,

Department of Clinical Neuroscience,

Karolinska Institutet, Stockholm, Sweden

Email: Korinna.karampampa@ki.se

For peer review only

Abstract

Objectives: Telephone advice nursing is introduced in many countries with one key aim being a reduction of avoidable healthcare visits. The aim of this study was to explore whether callers to a telephone advice nursing service followed the telenurses' recommendations, and whether there was a change in the level and trend of the rate of healthcare visits after the introduction of telephone advice nursing.

Design: Observational study.

Setting: Primary and secondary care in Jönköping Region, Sweden.

Participants: Telephone advice nursing calls, 06-23 hours, 2014-2015 (n=185,994) and outpatient healthcare visits 2012-2015 (n=6,877,266).

Primary outcome: Proportion of callers who visited healthcare within the time period advised by the telenurse.

Secondary outcome: Change in level or trend of the overall rate of healthcare visits per 1,000 persons and four-week period after the introduction of telephone advice nursing, with subgroup analysis for primary and secondary care.

Results: 77 % of callers who were recommended either to visit healthcare within 24 hours or to 'wait and see' followed the recommendations. There was no significant change in level (-5.15; 95% CI -15.80 to 5.50; p=0.349) or trend (-0.24; 95% CI -0.86 to 0.38; p=0.448) of the overall rate of visits per 1,000 persons and four-week period after the introduction of telephone advice nursing. For the rate of primary care visits, an increase in level (8.01; 95% CI 6.36 to 9.66; p<0.001) and trend (1.28; 95%

For peer review only

CI 1.17 to 1.39; *p*<0.001) were observed. For the rate of secondary care visits, a decrease in level (-8.77, 95% CI -14.41 to -3.13; *p*=0.004) and trend (-1.03, 95% CI -1.35 to -0.71; *p*<0.001) were observed.

Conclusions: The introduction of telephone advice nursing may have contributed to a shift in the rate of healthcare visits from secondary to primary care.

Keywords: Telephone advice nursing, Telenursing, telephone advice nursing, Telehealth, Interrupted time series analysis

Strengths and limitations of this study

- *Observational study based on patient-level, registry data covering four years and over 7 million observations.*
- *Statistical analysis using interrupted time series analysis with segmented regression, a recommended research design when randomisation is not possible.*
- *No control series was used to control for potential confounders in the interrupted time series analysis.*
- *Results might not be generalizable to the rest of Sweden, or to other countries.*

For peer review only

Background

A striking development around the Western world is the increased availability of telephone triage and advice services (henceforth telephone advice nursing), where telephone nurses (henceforth telenurses) provide callers with triage, advice, support, education, referral to adequate care levels, healthcare information and coordinate healthcare resources.[1] Telephone advice nursing has been implemented in the United Kingdom, Denmark, Sweden, Australia, the USA, the Netherlands and Canada.[2–4] In Sweden, the telephone advice nursing service 1177 Vårdguiden (henceforth 1177) was implemented successively across the regions between 2003-2013.[5] The service has the five minimum components required to ensure patient safety: qualified staff, medically approved guidelines, electronic medical records, staff training programmes and standards.[6,7]

One key aim from policymakers when implementing telephone advice nursing services is helping care seekers find the appropriate level of care and reducing unnecessary healthcare visits.[8,4,9,10] Swedish healthcare providers hypothesised that 1177 would contribute to a more efficient healthcare system.[11–13] It was predicted that if callers followed the telenurses' recommendations, the service would impact upon the rates of healthcare visits as a greater proportion of patients would be able to self-care or seek care at more appropriate healthcare levels than they would without access to telephone advice nursing.[11] The anticipated effect from this transition was an overall reduction of costly emergency visits to secondary care, which would free resources in the healthcare system. The rate of primary care visits was expected to remain more or less unchanged but with a more appropriate case mix.[11]

For peer review only

The available evidence worldwide regarding the extent to which callers follow recommendations from telenurses and telephone advice nursing's impact on healthcare visits is inconsistent.[2,4,8,10] Context-specific factors such as healthcare organization and workplace environment affect the external validity of findings.[8,14] Identifying key policy lessons for any given telephone advice nursing service therefore requires it to be studied specifically.

For 1177, evidence regarding the extent to which callers follow recommendations from telenurses is diverse. 1177's own surveys to callers find that 90 % follow the recommendations,[15] while a government agency used observational data to find that 55 – 78 % follow recommendations depending on the advice provided.[16]

Similarly, evidence regarding 1177's impact on the rate of healthcare visits is scarce. Four studies have found telephone advice nursing services in Sweden to redistribute visits to less costly levels of care.[17–20] Two of the studies concerned local predecessors to 1177.[17,18] All studies based their conclusions on self-reported survey data rather than observational data, which possibly limits the strength of the evidence.

To explore the hypothesis that 1177 may have freed resources in the healthcare system through reducing avoidable healthcare visits, the aim of this study was to use observational data to evaluate whether 1177 callers follow the telenurses' recommendations, and whether there was any change in the level and trend of healthcare visits after the introduction of telephone advice nursing.

For peer review only

For peer review only - http://bmjopen.bmj.com/site/about/guidelines.xhtml

Methods

Study design and setting

This was an observational study which used patient-level data from the patient administrative system in Jönköping Region, Sweden. The region manages a tax-financed healthcare system with universal coverage for its 345,000 inhabitants.[21–24] 1177 was made available to all inhabitants in the region on September 2nd 2013. Before that, telephone advice nursing was provided by individual hospitals and health care centres in the region. When 1177 was introduced, seven regional telephone advice lines were closed, including those to emergency departments and out-of-hours centres. No other large changes to healthcare in the region were carried out in 2012-2015.[21–24]

In contrast to previous advice lines, 1177 was always open and staffed with nurses specifically trained in telephone advice nursing.[7] The service followed national standards in telephone advice nursing, with a key component being consistent use of the national symptom-based computer decision support system (CDSS). The CDSS contained symptoms with related urgency levels, as well as a referral catalogue of available health care services with opening times, addresses as well as any specific rules about which patient groups different healthcare units receive. The nurses identified the callers' main symptom and thereby obtained the urgency level from the CDSS. They thereafter used the catalogue in combination with their knowledge of the healthcare organization to find care that would meet the callers' needs within the urgency level, given factors such as opening times, geography and accessibility. The region intended 1177 to be a first point of contact with healthcare for inhabitants, along with the options

For peer review only

of reading information on the 1177 website, calling a healthcare centre or visiting an emergency department or out-of-hours care centre.[25]

To determine the percentage of callers who follow the recommendations of the telenurse, calls to 1177 were linked to information on any subsequent outpatient healthcare visits (emergency visits, visits that required referrals and visits that did not require referrals). The linkage and subsequent anonymization were performed by the system administrator using the unique personal identity number that Swedish residents have. Only anonymized patient-level data was used in the study.

To examine whether there was any change in the level and trend of healthcare visits after 1177 was introduced, anonymized patient-level data on all outpatient healthcare visits in the region (emergency visits, visits that required referrals and visits that did not require referrals) was used.

The study was approved by the Swedish Ethical Review Authority (2019-01784).

Definitions and statistical analysis

The first study outcome of whether callers followed recommendations of the telenurse was defined as the proportion of callers who visited healthcare within the time period advised by the telenurse.

To evaluate this outcome, calls to 1177 between 6 am and 11 pm and any subsequent healthcare visit at any time of day, that took place within 72 hours of the call between January 1st 2014 and December 31st 2015 (n = 185,994) were used. Calls that referred patients to healthcare outside of the region were excluded (n = 1,839). Night-time calls

For peer review only

between 11 pm and 6 am were unavailable for study as they were answered by a neighbouring county council on behalf of Jönköping Region.

Variables included date and time of call, callers' age and sex, the 'urgency level', as well as the name of any healthcare unit visited within 72 hours of the call, and if the visit occurred within 24 hours.

The variable 'urgency level' described how urgent the telenurse found it for the caller to visit healthcare. The variable had five different levels: 'immediately', 'quickly' (meaning 'within a few hours'), 'within 24 hours', 'a weekday in near time' (meaning within a week or a few weeks) and 'wait and see' (meaning no need to visit healthcare). These five levels were collapsed into three levels, since the data did not allow following the caller's subsequent behaviour on a more granular level:

- Visit within 24 hours (including 'immediately', 'quickly' and 'within 24 hours')
- Visit within a few weeks (including 'a weekday in near time')
- 'Wait and see' (including 'wait and see')

Each call was mapped to the data on healthcare visits, providing the proportion of callers that were recommended to visit within 24 hours and did so, as well as the proportion of callers recommended to 'wait and see' that did not visit healthcare within 72 hours of the call. The proportion of callers who followed the recommendation to visit healthcare within a few weeks could not be determined as the data on healthcare visits only stretched to 72 hours after the call.

For peer review only

180 To evaluate the second study outcome, i.e. to determine whether the rate of healthcare visits changed after the introduction of 1177, all outpatient healthcare visits between January 23, 2012 and December 20, 2015 were used (n=6,877,266). Visits made by patients not registered as residents in the region were excluded (n=215,471). Interrupted time series with segmented regression was used to study this outcome, because it is a robust statistical method used where randomised controlled trials cannot be performed.[26–29] The central assumption of the method was that the trend of healthcare visits before the introduction of telephone advice nursing would have continued unchanged if the service had not been introduced.

189 The data covered 51 four-week periods; 21 before 1177 was implemented and 30 after. Variables included date and time of the visit. The series' data points were calculated as visits per 1,000 population and four-week period. The rates were controlled for population growth by using the population size for each given year as the denominator.

194 Three time series were made, one for all outpatient visits (primary and secondary care) and one for each subgroup of outpatient primary and secondary care visits. To create the subgroups for outpatient primary and secondary care, the healthcare units were categorised according to a definition from the Swedish Agency for Health and Care Services:[16]

199 • Primary care: primary care centres, physicians' centres, out-of-hours primary care, children's healthcare centres and maternal healthcare centres.
201 • Secondary care: specific or hospital-based specialist care including emergency care.

For peer review only

Regression models with two segments were fitted to the data, one for the period pre-1177 and one for the period after. Separate levels (intercepts) and trends (slopes) were estimated for each segment.[27] Statistical tests were carried out to estimate changes in the level or trend after the introduction of 1177. To estimate the size of the effect, the models were used to calculate the rates one year (12 four-week periods) after 1177 was introduced and the relative changes compared to the rates that would have been if the trend before 1177 was introduced had continued unchanged.

To control for seasonality, harmonic sine and cosine pairs (‘Fourier terms’) were added to the model.[30] To control for autocorrelation, a moving averages term of order q=13 was added using generalized least squares regression with a maximum likelihood method. Three plausible alternative autocorrelation terms were tested using likelihood-ratio tests. Since the Akaike information criterion did not indicate improvements to the fit of the model, q=13 was retained. Nevertheless, some autocorrelation remained.

Patient and public involvement

Patients and public were not involved in the design and conduct of this study.

For peer review only

For peer review only - http://bmjopen.bmj.com/site/about/guidelines.xhtml

Results

Calls to the telephone advice nursing service represented 5 % of outpatient contacts. The proportion that followed the recommendation differed between groups (Table 1). A majority of those recommended to visit healthcare within 24 hours did so (73 %), and 84 % of those recommended to 'wait and see' did not visit healthcare within 72 hours. Combining these groups, 77 % followed the recommendation. When stratified by age groups and sex, the proportion that followed the telenurses' recommendation varied between 66 % (females aged 20-39) and 90 % (children aged 0-9).

Table 1. Callers that followed the recommendation to visit healthcare or 'wait and see', Jönköping Region 2014-2015.

		Followed the recommendation to visit healthcare within 24 h n(%)		Followed the recommendation to 'wait and see' n(%)		Both groups combined n(%)	
	All (n=162,614)	75,159	(73)	50,836	(84)	125,995	(77)
	Males (n=72,180)	34,403	(75)	22,533	(86)	56,936	(79)
	Females (n=90,434)	40,756	(72)	28,303	(83)	69,059	(76)
All	Age group	Followed the recommendation to visit healthcare within 24 h n(%)		Followed the recommend-action to 'wait and see' n(%)		Both groups combined n(%)	
	0-9 (n=51,412)	18,925	(70)	22,045	(90)	40,970	(80)
	10-19 (n=16,032)	8,559	(79)	4,388	(84)	12,947	(81)
	20-39 (n=43,489)	18,605	(68)	13,273	(82)	31,878	(73)
	40-59 (n=25,289)	13,972	(77)	5,624	(80)	19,596	(77)
	>60 (n=26,392)	15,098	(79)	5,505	(77)	20,603	(78)
Males	Age group						
	0-9 (n=27,562)	10,246	(71)	11,769	(90)	22,015	(80)
	10-19 (n=7,365)	4,065	(81)	2,857	(84)	6,044	(82)
	20-39 (n=15,488)	7,232	(71)	4,496	(84)	11,728	(76)
	40-59 (n=10,706)	6,223	(78)	2,207	(80)	8,430	(79)
	>60 (n=11,059)	6,637	(80)	2,082	(76)	8,719	(79)
Females	Age group						
	0-9 (n=23,850)	8,679	(70)	10,276	(90)	18,955	(79)
	10-19 (n=8,667)	4,494	(78)	2,409	(83)	6,903	(80)
	20-39 (n=28,001)	11,373	(66)	8,777	(81)	20,150	(72)
	40-59 (n=14,583)	7,749	(76)	3,417	(79)	11,166	(77)
	>60 (n=15,333)	8,461	(78)	3,423	(77)	11,884	(78)

For peer review only

For peer review only - http://bmjopen.bmj.com/site/about/guidelines.xhtml

Overall, for all healthcare visits, the final segmented regression model showed no significant changes in level (-5.15; 95% CI -15.8 to 5.5; *p*=0.349) or trend (-0.24; 95% CI -0.86 to 0.38; *p*=0.448) of the healthcare visits after the introduction of 1177. Statistically significant changes after the introduction of 1177 were found only in the subgroup analysis. Immediately after the introduction of 1177, primary care visits had an increase of 8.01 visits per 1,000 persons (95% CI 6.36 to 9.66; *p*<0.001) and had a trend increase of 1.28 visits per 1,000 per four-week period (95% CI 1.17 to 1.39; *p*<0.001). In contrast, visits to secondary care decreased with 8.77 visits per 1,000 weeks immediately after the introduction of telephone advice nursing (95% CI -14.41 to -3.13; *p*=0.004), and had a trend decrease of 1.03 visits per 1,000 per four week period (% CI -1.35 to -0.71; *p*<0.001) (Table 2 and Figure 1).

When the regression models were used to estimate the effect size one year (12 four-week periods) after the introduction of 1177, an 11.5 % increase in the rate of primary care visits and an 11 % decrease in the rate of secondary care visits was found (Table 3).

Table 2. Results of the interrupted time series analysis for healthcare visits, Jönköping Region 2012-2015.

	Level (intercept)	Trend (slope)	Change in level after telephone advice nursing	Change in trend after telephone advice nursing
All visits (primary and secondary care)				
Co-efficient	376.909	0.769	-5.152	-0.244
95% CI	368.501 to 385.317	0.058 to 1.480	-15.797 to 5.497	-0.863 to 0.383
p-value	<0.001	0.040	0.349	0.448
Primary care visits				
Co-efficient	240.49	-1.164	8.013	1.279
95% CI	238.738 to 242.242	-1.297 to -1.031	6.36 to 9.66	1.17 to 1.39
p-value	<0.001	<0.001	<0.001	<0.001
Secondary care visits				

For peer review only

Co-efficient	142.861	1.437	-8.767	-1.031
95% CI	138.594 to 147.128	1.074 to 1.8	-14.413 to -3.127	-1.349 to -0.711
p-value	<0.001	<0.001	0.004	<0.001

Figure 1. Interrupted time series analysis for the rate of healthcare visits, Jönköping Region 2012-2015. (a) All visits (primary and secondary care). (b) Primary care visits. (c) Secondary care visits. [1]

-------------Insert Figure 1-------------

Table 3. Observed rates of healthcare visits 12 four-week periods after telephone advice nursing was introduced and rates if the trends observed pre-implementation would have continued unchanged.

	All visits (primary and secondary care) (p=0.448)	Primary care visits (p<0.001)	Secondary care visits (p<0.001
Observed rate per 1,000 persons	394.20	225.59	169.13
Rate per 1,000 persons if the trend observed pre-implementation would have continued unchanged	402.28	202.23	190.27
Absolute change (observed rate– rate if the trend observed pre-implementation would have continued unchanged)	-8.02	23.36	-21.14
Relative change	-2.01 %	11.55 %	-11.11%

For peer review only

[1] In these graphical displays of the interrupted time series analyses, the trends of the rates of health care visits pre- and post- implementation of 1177 are shown (the thick solid lines). The pre-implementation trend has been extrapolated to the post-implementation period (the thick dotted line), to show what would have happened if the pre-implementation trends would have continued unchanged. The data points used for the analysis are shown in grey. The model fitted to the data show the seasonal trends (the thin grey lines).

Discussion

To summarize, the findings of this study indicate that large proportions (77%) of those who call 1177 follow the recommendations, and that there was in a shift in healthcare visits from secondary to primary care after the introduction of 1177. It is possible that the introduction of 1177 contributed to these changes, enabling more patients to seek care at lower levels than they could prior to the service's introduction.

The observed changes in the rate of healthcare visits broadly support the findings of previous survey-based studies in Sweden, which have suggested that telephone advice nursing services in the Swedish setting reduce emergency visits.[17–19] The interpretation that 1177 contributed to the observed changes in the rates of healthcare visits is also strengthened by the lack of plausible alternative explanations: the supply of primary and secondary care was stable throughout the study period and no other potential confounders were found.[21–24]

Compared to 1177's own survey-based findings on the proportions that follow the recommendations of the telenurse (90 %), 77 % is lower.[15] This is also the case when considering earlier survey-based studies on local predecessors to 1177.[1,17,18,31] However, the finding is similar to those of a government agency report that used observational data.[16] This contrast between survey data and observational data is supported by systematic reviews of the international literature on telephone advice nursing, according to which self-reported data tends to provide higher proportions than observational data.[2]

For peer review only

For peer review only - http://bmjopen.bmj.com/site/about/guidelines.xhtml

These inconsistent results of survey and observational data respectively may be considered as complementary as they to some extent measure different constructs. While observational studies require researchers to define what it means to follow the recommendations of the telenurse, survey-based studies let respondents interpret its meaning subjectively.[32] Although the latter study design suffers from threats to validity, primarily from recall-, social desirability- and nonresponse bias,[33–35] it also allows for capturing nuances of what it means to follow recommendations, and for studying the patient's perspective.

There are a broad range of possible care-seeking scenarios concealed behind the proportions found in this study. First, when callers appear to have followed the recommendations, it is not certain that the call to 1177 were what led them to do what they did. They may have based their decisions on consultations with primary care, family and friends, websites, and so forth. Second, the data in this study only showed the telenurse's main recommendation and cannot reflect potential alternative courses of action that the telenurse and caller may have also discussed, for example depending on how the patient's health status developed. Third, care sought after a call to 1177 within the time recommended by the nurse may have been for a separate issue than that discussed with the telenurse. Fourth, care recommended by the telenurse may have been inaccessible to the caller, making it impossible to follow the recommendations. Finally, callers may have chosen not to follow the recommendations because they lacked confidence in the telenurse or found the recommendations inappropriate.[20,36,37]

For peer review only

For peer review only - http://bmjopen.bmj.com/site/about/guidelines.xhtml

Strengths and limitations of the study

The strength of the study is that complete anonymized patient-level data was used, providing a strong design for answering the research questions. Unlike previous studies, this study's findings were derived from a large number of observations, free from the biases of surveys.[33] The interrupted time series analysis was the next-best statistical method for studying the rate of healthcare visits before and after the introduction of 1177 given a randomised, controlled trial was not possible.[27–29]

This study also has several limitations. Ideally, a control series should have been used to control for potential confounders.[27] In lack of a control group, media archives and grey literature were searched to identify potential confounding events, but none were found.[21–24]

The autocorrelation that remained in the segmented regression model fitted to the data, despite attempts to control for it, is another limitation. Autocorrelation does not affect coefficients, but it does affect the standard errors, which means we cannot rely on the p-values. This is particularly the case when p-values are close to 0.05. In the results of this study, however, p-values for most statistically significant results were <0.001.

Finally, a limitation concerns the generalizability of the results. First, this study has focused on 1177 in one Swedish region. While the healthcare organization across the regions in Sweden do not differ substantially, some differences may be present regarding the availability of healthcare services and the population demographics. Therefore, the results might not be generalizable to the rest of Sweden, or to other countries.

For peer review only

In addition, due to the unavailability of data, night calls during the hours 11pm and 6am were excluded from this study. However, it is unlikely that the behaviour of night-time callers would be substantially different than that of people calling during the rest of the day. Still, since the level of urgency may be different between e.g. morning or night calls, the results of this study may be not generalizable to calls/callers throughout the entire day.

Implications for future research and policy

For policy makers, this study provides some support that the implementation of telephone advice nursing contributed to their aims of freeing resources in the healthcare system, as a large proportion of callers followed the nurses' recommendations, and as the service may have contributed to the change in the rate of healthcare visits. The implication for policy is that telephone advice nursing might be considered as a positive element in the healthcare organisation.

Three areas should be of specific interest to future research and policy. One is the effects of technology used in telephone advice nursing: given the rapid technological developments such as automatic triage and the use of photos and video calls in telephone advice nursing, it should be of interest to find out more about how technology is being used and can be used to develop and enhance telephone advice nursing and its role in the healthcare system.[8]

Another area to further study is how the interaction between telephone advice nursing and other services in the healthcare system affect efficiency. For example, studies have established that one of the largest challenges experienced by telenurses at 1177 in

For peer review only

Sweden is the lack of adequate resources in the healthcare organisation, with insufficient availability of primary care being the largest problem.[3,6,38–43] A recent study has also shown that the healthcare organisation is not adapted to meet the needs of callers with mental illness.[44] If the healthcare organisation is not set up to meet the needs of the population, 1177 cannot contribute to system efficiency with its full potential. Future studies of 1177's contribution to system efficiency should treat it not as an isolated service but as one whose efficacy is interlinked with the functioning of the healthcare system as a whole.[45]

In addition, future research may focus on how access to, and use of, telephone advice nursing varies in the population. This study found that only 5 % of outpatient healthcare contacts were calls to 1177, raising the question of whether more patients could benefit from using the service. This aspect has not been studied in Sweden, but studies in the United Kingdom have found that the former telephone advice nursing service National Health Service Direct was used more by some groups. Important determinants were ethnicity, gender, age and socio-economic status.[46–49] If telephone advice nursing can contribute to inhabitants receiving care at the most appropriate levels, it is important that all groups gain equal access to the service.

Conclusions

The results indicate that large proportions of those who call 1177 follow the recommendations and that the introduction of 1177 in Region Jönköping may have contributed to a shift in healthcare visits from secondary to primary care. The results provide some support to the hypothesis that 1177 may have contributed to system efficiency, freeing resources through reducing unnecessary costly healthcare visits.

For peer review only

For peer review only - http://bmjopen.bmj.com/site/about/guidelines.xhtml

Declarations

Ethics approval and consent to participate

Informed consent was not collected from the participants of this study. The study was approved by the Swedish Ethical Review Authority, registered under number 2019-01784.

Availability of data and materials

The data that support the findings of this study are available from Region Jönköping, but restrictions apply to the availability of these data, which were used under license for the current study, and so are not publicly available.

Competing interests

AS is employed by Inera AB, a limited company owned by Swedish regions, municipalities as well as the Swedish Association of Local Authorities and Regions (SALAR) that administrates the technical solutions used in 1177. AS conducted this research as part of her master's degree project in the Master's programme in Public Health Sciences (track Health Economics, Policy and Management) at the Karolinska Institutet, while she was on leave of absence from her employment. The study findings have been interpreted scientifically by all co-authors, without the involvement of Inera AB, and not in the interest of 1177.

All other co-authors (ACW, NZ, and KK) are employed at or affiliated with Karolinska Institutet, Stockholm, Sweden.

For peer review only

KK is currently employed by BMS, a pharmaceutical company which is not involved with 1177 in Sweden.

Funding

This research received no specific grant from any funding agency in the public, commercial or not-for-profit sectors.

Authors' Contributions

AS, NZ and KK contributed to the conceptualization of the research questions, study design and methods. AS performed the analysis of the data, interpreted the study findings, and drafted the manuscript. All remaining authors, AS, NZ, KK and ACW assisted in the interpretation of the study findings and contributed to the manuscript with comments, suggestions and text.

Acknowledgements

Not applicable.

For peer review only

References

1 Wahlberg AC. Telephone advice nursing: callers' perceptions, nurses' experience of problems and basis for assessments. 2004.

2 Lake R, Georgiou A, Li J, et al. The quality, safety and governance of telephone triage and advice services – an overview of evidence from systematic reviews. BMC Health Serv Res 2017;**17**. doi:10.1186/s12913-017-2564-x

3 Wahlberg AC, Bjorkman A. Expert in nursing care but sometimes disrespected- Telenurses' reflections on their work environment and nursing care. J Clin Nurs 2018;**27**:4203–11. doi:10.1111/jocn.14622

4 Gidora H, Borycki EM, Kushniruk AW. Effects of Telenursing Triage and Advice on Healthcare Costs and Resource Use. Stud Health Technol Inform 2019;**257**:133–9.

5 Leppänen V. Inledning. In: Telefonrådgivning inom hälso- och sjukvård. Hungary: : Studentlitteratur 2008.

6 Wheeler SQ, Greenberg ME, Mahlmeister L, et al. Safety of Clinical and Non-Clinical Decision Makers in Telephone Triage: A Narrative Review. J Telemed Telecare 2015;**21**:305–22. doi:10.1177/1357633X15571650

7 Inera. 1177 Vårdguiden på telefon - Inera. 2021.https://www.inera.se/tjanster/1177-vardguiden/1177-vardguiden-pa-telefon/ (accessed 24 Jun 2021).

8 Boggan JC, Shoup JP, Whited JD, et al. Effectiveness of Acute Care Remote Triage Systems: a Systematic Review. J Gen Intern Med 2020;**35**:2136–45. doi:10.1007/s11606-019-05585-4

9 Campbell JL, Fletcher E, Britten N, et al. Telephone triage for management of same-day consultation requests in general practice (the ESTEEM trial): a cluster-randomised controlled trial and cost-consequence analysis. Lancet 2014;**384**:1859–68. doi:10.1016/S0140-6736(14)61058-8

10 Ismail SA, Gibbons DC, Gnani S. Reducing inappropriate accident and emergency department attendances: a systematic review of primary care service interventions. Br J Gen Pr 2013;**63**:e813-20. doi:10.3399/bjgp13X675395

11 Landstingsförbundet. Vårdråd direkt - Sjukvårdsrådgivningar i samverkan. Slutrapport från utredningen om nationellt samordnad sjukvårdsrådgivning. Stockholm: : Landstingsförbundet 2003.

12 Stienstedt G. Effektiv vård: slutbetänkande av en nationell samordnare för effektivare resursutnyttjande inom hälso- och sjukvården. Stockholm: : Statens Offentliga Utredningar 2016.

For peer review only

13 Kaminsky E, Röing M, Björkman A, et al. Telephone nursing in Sweden: A narrative literature review. Nurs Health Sci 2017;**19**:278–86. doi:10.1111/nhs.12349

14 Lewinski AA, Rushton S, Van Voorhees E, et al. Implementing remote triage in large health systems: A qualitative evidence synthesis. Res Nurs Health 2021;**44**:138–54. doi:10.1002/nur.22093

15 Eklöf P. 1177 Vårdguiden på telefon. 2017.https://www.1177.se/Om-1177/1177-sjukvardsradgivning/ (accessed 12 Jan 2018).

16 En primär angelägenhet - kunskapsunderlag för en stärkt primärvård med patienten i centrum. Myndigheten för vård- och omsorgsanalys 2017.

17 Nordling S. Ekonomisk utvärdering av sjukvårdsupplysning per telefon i Jämtland och Malmö. Lund: : IHE 2001.

18 Marklund B, Ström M, Månsson J, et al. Computer-supported telephone nurse triage: an evaluation of medical quality and costs. J Nurs Manag 2007;**15**:180–7. doi:10.1111/j.1365-2834.2007.00659.x

19 Rahmqvist M, Husberg M. Effekter av sjukvårdsrådgivning per telefon - en analys av rådgivningsverksamheten 1177 i Östergötland och Jämtland. Linköping: : Linköpings universitet, Centrum för utvärdering av medicinsk teknologi 2009.

20 Martinsson J, Gustafsson S. Modeling the effects of telephone nursing on healthcare utilization. Int J Med Inf 2018;**113**:98–105. doi:10.1016/j.ijmedinf.2018.02.004

21 Region Jönköpings län. Årsredovisning 2015: med förvaltningsberättelse. 2016.

22 Region Jönköpings län. Årsredovisning 2014: med förvaltningsberättelse. 2015.

23 Landstinget i Jönköpings län. Årsredovisning 2012: med förvaltningsberättelse. Huskvarna: 2013.

24 Landstinget i Jönköpings län. Årsredovisning 2013: med förvaltningsberättelse. Ängelholm: 2014.

25 Landstingsstyrelsen, Landstinget i Jönköpings län. Införande av 1177 och ett gemensamt kundcenter. 2012.

26 Kontopantelis E, Doran T, Springate DA, et al. Regression based quasi-experimental approach when randomisation is not an option: interrupted time series analysis. BMJ 2015;**350**:h2750. doi:10.1136/bmj.h2750

27 Shadish WR, Cook TD, Campbell DT. Experimental and Quasi-Experimental Designs for Generalized Causal Inference. Belmont, California: : Wadsworth Cengage Learning 2002.

For peer review only

For peer review only - http://bmjopen.bmj.com/site/about/guidelines.xhtml

28 Bernal JL, Cummins S, Gasparrini A. Interrupted time series regression for the evaluation of public health interventions: a tutorial. Int J Epidemiol 2017;**46**:348–55. doi:10.1093/ije/dyw098

29 Penfold RB, Zhang F. Use of Interrupted Time Series Analysis in Evaluating Health Care Quality Improvements. Acad Pediatr 2013;**13**:S38–44. doi:10.1016/j.acap.2013.08.002

30 Bhaskaran K, Gasparrini A, Hajat S, et al. Time series regression studies in environmental epidemiology. Int J Epidemiol 2013;**42**:1187–95. doi:10.1093/ije/dyt092

31 Wahlberg AC, Wredling R. Telephone nursing: Calls and caller satisfaction. Int J Nurs Pract;**5**:164–70. doi:10.1046/j.1440-172x.1999.00179.x

32 Gustafsson S, Martinsson J, Wälivaara B-M, et al. Influence of self-care advice on patient satisfaction and healthcare utilization. J Adv Nurs 2016;**72**:1789–99. doi:10.1111/jan.12950

33 Hennekens CH, Buring JE, Mayrent SL. Epidemiology in Medicine. First edition. United States of America: 1987.

34 Krumpal I. Determinants of social desirability bias in sensitive surveys: a literature review. Qual Quant 2013;**47**:2025–47. doi:10.1007/s11135-011-9640-9

35 Gorman E, Leyland AH, McCartney G, et al. Assessing the Representativeness of Population-Sampled Health Surveys Through Linkage to Administrative Data on Alcohol-Related Outcomes. Am J Epidemiol 2014;**180**:941–8. doi:10.1093/aje/kwu207

36 Kaminsky E, Carlsson M, Röing M, et al. ‘If I didn’t trust Swedish Healthcare Direct, I would never call’ – views of making pediatric health calls. Clin Nurs Stud 2013;**1**:57. doi:10.5430/cns.v1n3p57

37 Winneby E, Flensner G, Rudolfsson G. Feeling rejected or invited: Experiences of persons seeking care advice at the Swedish Healthcare Direct organization. Jpn J Nurs Sci 2014;**11**:87–93. doi:10.1111/jjns.12007

38 Wahlberg AC, Cedersund E, Wredling R. Bases for assessments made by telephone advice nurses. J Telemed Telecare 2005;**11**:403–7. doi:10.1177/1357633X0501100805

39 Bjorkman A, Engstrom M, Olsson A, et al. Identified obstacles and prerequisites in telenurses’ work environment - a modified Delphi study. BMC Health Serv Res 2017;**17**:357. doi:10.1186/s12913-017-2296-y

40 Holmström I, Dall’Alba G. `Carer and gatekeeper’ – conflicting demands in nurses’ experiences of telephone advisory services. Scand J Caring Sci 2002;**16**:142–8. doi:10.1046/j.1471-6712.2002.00075.x

For peer review only

41 Wahlberg AC, Cedersund E, Wredling R. Telephone nurses' experience of problems with telephone advice in Sweden. J Clin Nurs 2003;**12**:37–45. doi:10.1046/j.1365-2702.2003.00702.x

42 Holmström I, Höglund AT. The faceless encounter: ethical dilemmas in telephone nursing. J Clin Nurs 2007;**16**:1865–71. doi:10.1111/j.1365-2702.2007.01839.x

43 Ernesäter A, Engström M, Holmström I, et al. Incident reporting in nurse-led national telephone triage in Sweden: the reported errors reveal a pattern that needs to be broken. J Telemed Telecare 2010;**16**:243–7. doi:10.1258/jtt.2009.090813

44 Bjorkman A, Salzmann-Erikson M. When all other doors are closed: Telenurses' experiences of encountering care seekers with mental illnesses. Int J Ment Health Nurs 2018;**27**:1392–400. doi:10.1111/inm.12438

45 Björkman A, Engström M, Winblad U, et al. Malpractice claimed calls within the Swedish Healthcare Direct: a descriptive - comparative case study. BMC Nurs 2021;**20**:21. doi:10.1186/s12912-021-00540-3

46 Bibi M, Attwell RW, Fairhurst RJ, et al. The usage of NHS Direct by different ethnic and gender groups in an urban population. Rev Env Health 2008;**23**:235–41.

47 Shah SM, Cook DG. Socio-economic determinants of casualty and NHS Direct use. J Public Health Oxf 2008;**30**:75–81. doi:10.1093/pubmed/fdn001

48 Cook EJ, Randhawa G, Large S, et al. A U.K. case study of who uses NHS direct: investigating the impact of age, gender, and deprivation on the utilization of NHS direct. Telemed J E Health 2012;**18**:693–8. doi:10.1089/tmj.2011.0256

49 Cook EJ, Randhawa G, Large S, et al. Who uses NHS Direct? Investigating the impact of ethnicity on the uptake of telephone based healthcare. Int J Equity Health 2014;**13**:99. doi:10.1186/s12939-014-0099-x

For peer review only

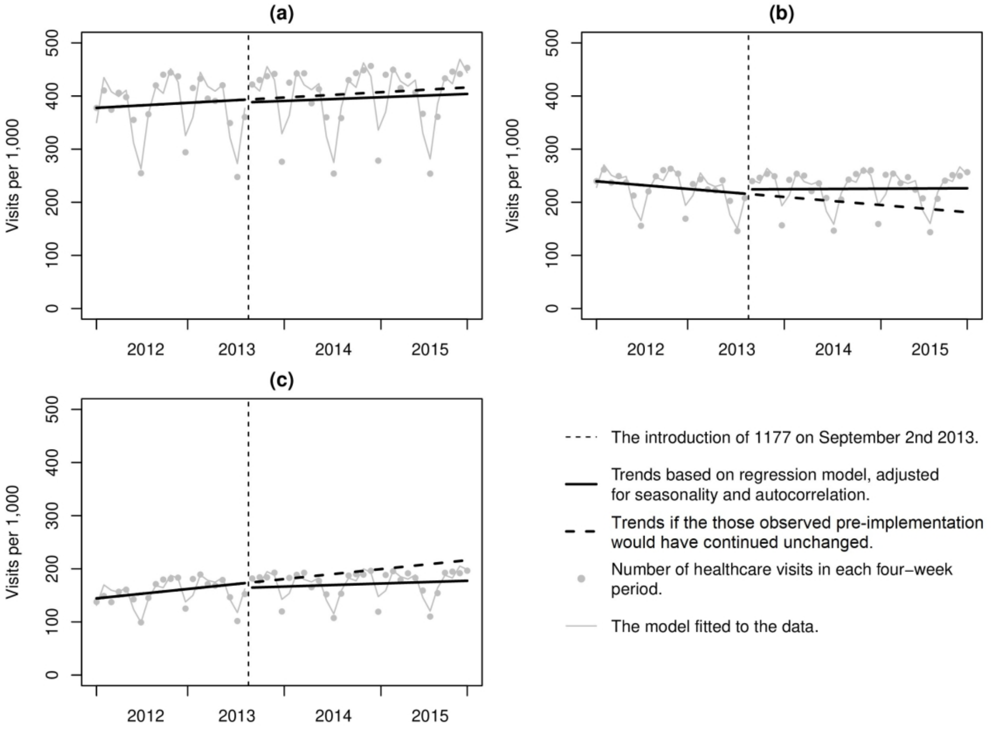

661x484mm (96 x 96 DPI)

STROBE 2007 (v4) Statement—Checklist of items that should be included in reports of *cohort studies*

Section/Topic	Item #	Recommendation	Reported on page #
**Title and abstract**	1	(*a*) Indicate the study's design with a commonly used term in the title or the abstract	1
		(*b*) Provide in the abstract an informative and balanced summary of what was done and what was found	3-4
**Introduction**			
Background/rationale	2	Explain the scientific background and rationale for the investigation being reported	6-7
Objectives	3	State specific objectives, including any prespecified hypotheses	7
**Methods**			
Study design	4	Present key elements of study design early in the paper	8
Setting	5	Describe the setting, locations, and relevant dates, including periods of recruitment, exposure, follow-up, and data collection	8-11
Participants	6	(*a*) Give the eligibility criteria, and the sources and methods of selection of participants. Describe methods of follow-up	9-11
		(*b*) For matched studies, give matching criteria and number of exposed and unexposed	n.a.
Variables	7	Clearly define all outcomes, exposures, predictors, potential confounders, and effect modifiers. Give diagnostic criteria, if applicable	9-11
Data sources/ measurement	8*	For each variable of interest, give sources of data and details of methods of assessment (measurement). Describe comparability of assessment methods if there is more than one group	*9-11*
Bias	9	Describe any efforts to address potential sources of bias	11
Study size	10	Explain how the study size was arrived at	n.a.
Quantitative variables	11	Explain how quantitative variables were handled in the analyses. If applicable, describe which groupings were chosen and why	10-11
Statistical methods	12	(*a*) Describe all statistical methods, including those used to control for confounding	10-11
		(*b*) Describe any methods used to examine subgroups and interactions	10-11
		(*c*) Explain how missing data were addressed	9
		(*d*) If applicable, explain how loss to follow-up was addressed	n.a.
		(*e*) Describe any sensitivity analyses	n.a.
**Results**			

For peer review only

Participants	13*	(a) Report numbers of individuals at each stage of study—eg numbers potentially eligible, examined for eligibility, confirmed eligible, included in the study, completing follow-up, and analysed	12
		(b) Give reasons for non-participation at each stage	n.a.
		(c) Consider use of a flow diagram	n.a.
Descriptive data	14*	(a) Give characteristics of study participants (eg demographic, clinical, social) and information on exposures and potential confounders	12
		(b) Indicate number of participants with missing data for each variable of interest	9
		(c) Summarise follow-up time (eg, average and total amount)	12
Outcome data	15*	Report numbers of outcome events or summary measures over time	12-13
Main results	16	(*a*) Give unadjusted estimates and, if applicable, confounder-adjusted estimates and their precision (eg, 95% confidence interval). Make clear which confounders were adjusted for and why they were included	n.a.
		(*b*) Report category boundaries when continuous variables were categorized	12-13
		(*c*) If relevant, consider translating estimates of relative risk into absolute risk for a meaningful time period	n.a.
Other analyses	17	Report other analyses done—eg analyses of subgroups and interactions, and sensitivity analyses	12-14
**Discussion**			
Key results	18	Summarise key results with reference to study objectives	15
**Limitations**			
Interpretation	20	Give a cautious overall interpretation of results considering objectives, limitations, multiplicity of analyses, results from similar studies, and other relevant evidence	18-19
Generalisability	21	Discuss the generalisability (external validity) of the study results	20
**Other information**			
Funding	22	Give the source of funding and the role of the funders for the present study and, if applicable, for the original study on which the present article is based	21-22

*Give information separately for cases and controls in case-control studies and, if applicable, for exposed and unexposed groups in cohort and cross-sectional studies.

Note: An Explanation and Elaboration article discusses each checklist item and gives methodological background and published examples of transparent reporting. The STROBE checklist is best used in conjunction with this article (freely available on the Web sites of PLoS Medicine at http://www.plosmedicine.org/, Annals of Internal Medicine at http://www.annals.org/, and Epidemiology at http://www.epidem.com/). Information on the STROBE Initiative is available at www.strobe-statement.org.

For peer review only
